# Cross-docking and redocking reveal distinct determinants of success in physics-based and AI-driven binding pose prediction in protein–ligand complexes

**DOI:** 10.1039/d6ra05440d

**Published:** 2026-09-08

**Authors:** Kapali Suri, Anshul Yadav, Abhishek Tripathi, N. Arul Murugan

**Affiliations:** a Department of Computational Biology, Indraprastha Institute of Information Technology Okhla Industrial Estate Delhi 110020 India arul.murugan@iiitd.ac.in; b Centre for Excellence in Healthcare, Indraprastha Institute of Information Technology Okhla Industrial Estate Delhi 110020 India

## Abstract

Protein–ligand pose prediction is central to structure-based drug discovery, yet the relative performance of physics-based and AI-driven methods under realistic cross-docking conditions remains insufficiently characterized. Here, we compare physics-based docking methods (AutoDock4, AutoDock Vina, and DOCK 6) with data-driven approaches, including the deep-learning model GNINA 1.3 and the diffusion-based frameworks AlphaFold 3, Boltz-2, and DiffDock. Performance was evaluated using standardised redocking and cross-docking protocols across three Alzheimer's disease targets representing distinct binding-site architectures: acetylcholinesterase (AChE; deep gorge), β-secretase 1 (BACE1; flexible flap-controlled site), and glycogen synthase kinase-3β (GSK-3β; open, solvent-exposed pocket). Physics-based methods were competitive during redocking but showed substantial performance reductions under cross-docking, whereas diffusion-based approaches generally maintained higher cross-docking accuracy. GNINA 1.3 rigid achieved an 87.7% minimum heavy-atom RMSD success rate during redocking, which decreased to 13.5% during cross-docking, whereas AlphaFold 3, Boltz-2, and DiffDock achieved cross-docking success rates of 93.1%, 89.6%, and 85.7%, respectively. AlphaFold 3 consistently outperformed Boltz-2 despite its smaller training set, suggesting that predictive performance is influenced not only by training-data volume but also by factors such as model architecture and confidence calibration. Training-overlap analysis further showed that AI-based methods retained substantial failure rates even for complexes represented in their training data, indicating that training-data overlap alone does not ensure reliable pose prediction. Under the current protocol conditions, rigid docking outperformed flexible protocols, while flexible-docking pocket volumes showed more restricted sampling relative to experimental holo structures. Among the GNINA 1.3 configurations, CNN rescoring with refinement produced the highest pose-recovery success rates, followed by CNN rescoring alone and the default Vina/empirical scoring approach in cross-docking. Receptor conformational preference was target-dependent: holo structures provided higher docking accuracy for AChE and BACE1, whose ligand-bound cavities exhibited greater structural complexity and geometric confinement that favoured pose discrimination, whereas the apo GSK-3β structure contained a larger, more solvent-exposed cavity that improved ligand accessibility and docking performance. Overall, these findings demonstrate the importance of cross-docking and training-overlap-aware evaluation for assessing docking performance under realistic conditions and provide cavity-topology-based considerations for selecting docking strategies in structure-based drug discovery.

## Abstract 2

Protein–ligand pose prediction is central to structure-based drug discovery, yet the relative performance of physics-based and AI-driven methods under realistic cross-docking conditions remains insufficiently characterized.

## Graphical abstract

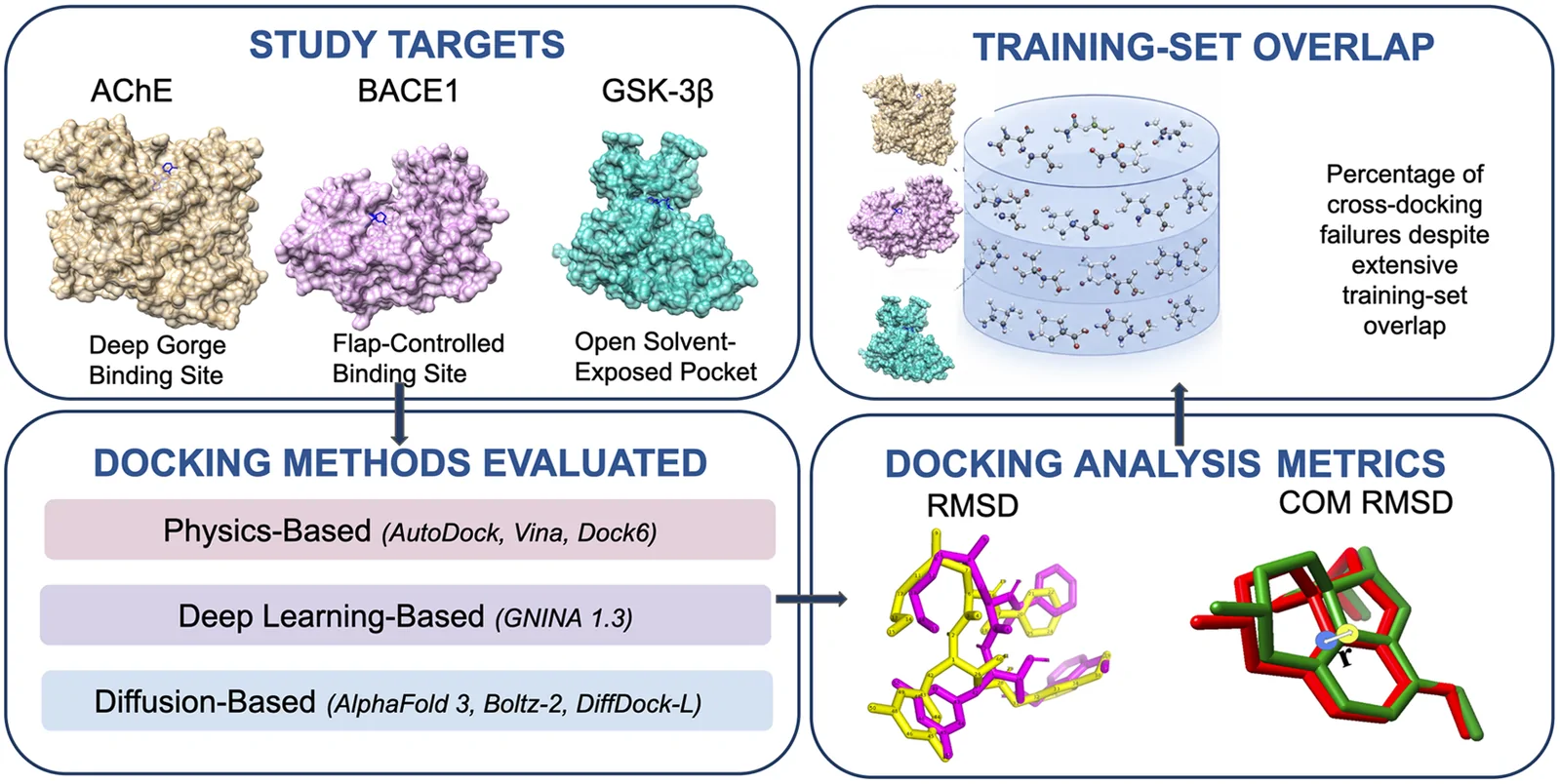

## Introduction

Protein–ligand interaction (PLI) prediction is a critical component of structure-based drug discovery and depends on multiple factors, including ligand geometry, physicochemical complementarity, and receptor conformational dynamics.^1^ Despite decades of methodological development, accurately predicting ligand-binding poses remains challenging, particularly for targets exhibiting substantial structural flexibility or ligand-induced conformational changes. These challenges are especially relevant in multifactorial diseases such as Alzheimer's disease (AD), where therapeutic targets encompass diverse functional classes and binding-site architectures.

Amyloid-β aggregation resulting from APP cleavage, cholinergic dysfunction caused by acetylcholine depletion, and dysregulated kinase signalling leading to tau hyperphosphorylation are key contributors to AD pathogenesis. Consequently, β-secretase 1 (BACE1), acetylcholinesterase (AChE), and glycogen synthase kinase-3β (GSK-3β) have emerged as important therapeutic targets for drug discovery.^2^ These proteins represent distinct binding-site architectures: AChE contains a deep, narrow gorge with multiple subpockets; BACE1 possesses a flap-regulated, partially enclosed cavity; and GSK-3β features an open, solvent-exposed pocket. This diversity provides an opportunity to investigate how binding-site geometry influences docking performance across both classical and AI-based methods. This aspect remains insufficiently explored in current benchmarking studies. Collectively, these targets provide a stringent benchmark because they encompass diverse ligand–recognition mechanisms and substantial apo–holo conformational variation. In this work, we systematically compare redocking and cross-docking strategies for BACE1, AChE, and GSK-3β under both rigid and flexible receptor conditions.

Physics-based docking methods, such as AutoDock4,^3,4^ AutoDock Vina,^5^ and DOCK 6,^6^ employ grid-based scoring functions that combine van der Waals, electrostatic, desolvation, and hydrogen-bonding terms with heuristic search algorithms to explore ligand conformational space (Table 1). Consequently, most large-scale docking studies rely on rigid receptor representations, implicitly assuming a static binding site. Although computationally efficient, this assumption can bias predictions toward crystallographic binding modes and obscure failures arising from receptor flexibility, cross-docking, or ligand-induced fit. Flexible docking partially addresses this limitation by allowing selected receptor residues and ligands to adapt during binding, thereby capturing aspects of induced-fit recognition.^7^ However, the resulting expansion of conformational search space substantially increases computational cost and complicates pose scoring, limiting its routine application and systematic evaluation.

**Table 1:** Overview of molecular docking protein–ligand structure prediction programs with technical specifications

Software	License	Version	Search/sampling algorithm	Protein flexibility support	Hardware support
**Physics-based**					
AutoDock Vina	Open source	1.2.7	Iterated local search (ILS) + Broyden–Fletcher–Goldfarb–Shanno (BFGS) optimization	Side-chain flexibility supported	CPU, GPU
AutoDock4	Open source	4.2.6	Lamarckian genetic algorithm (LGA) + solis–wets local search	Side-chain flexibility supported	CPU, GPU
DOCK 6	Open source	6.12	Anchor-and-Grow	Rigid receptor only	CPU

**Deep Learning**					
AlphaFold 3	Restricted open source	3	Diffusion-based generative model	Flexible receptor	GPU
Boltz-2	Open source	2	Reverse diffusion, PairFormer attention, Boltz steering	Only flexible receptor	CPU, GPU
GNINA 1.3	Open source	1.3	Vina sampling + CNN re-ranking	Side-chain flexibility supported	CPU, GPU
DiffDock	Open source	February 2024 release	Diffusion-based generative model	Rigid receptor only	GPU

These limitations have motivated the development of data-driven approaches. Diffusion-based frameworks such as AlphaFold 3,^8^ Boltz-2,^9^ and DiffDock,^10^ together with the deep-learning method GNINA 1.3,^11^ learn protein–ligand interaction patterns from large structural datasets and often improve pose ranking and blind-docking performance. Recent studies^12^ report substantial improvements in docking accuracy and virtual-screening efficiency, particularly when binding-site information is limited. Table 1 further contrasts these AI-based approaches with traditional docking methods in terms of algorithmic design, licensing, support for receptor flexibility, and computational requirements.

However, the extent to which these models generalise beyond previously observed protein–ligand systems remains unclear. AlphaFold 3, a diffusion-based framework that predicts protein–ligand complexes directly from amino-acid sequences, reported an 88.1% success rate with an average ligand heavy-atom root mean square deviation (HA-RMSD) of approximately 1.12 Å on coronavirus main proteases.^13^ Although these results are impressive, the extent to which AlphaFold 3 generalises to truly novel protein–ligand systems remains under active investigation.^14,15^ Furthermore, AlphaFold 3 has not been systematically compared with newer diffusion-based approaches, such as Boltz-2, in peer-reviewed benchmarks specifically focused on protein–ligand pose prediction.

Consequently, several important questions remain unresolved. First, it remains unclear whether the apparent superiority of diffusion-based methods over classical docking approaches reflects genuine structural generalisation or is influenced by training-data overlap. This uncertainty is compounded by the widespread use of redocking benchmarks, which evaluate ligands in their cognate receptor conformations and may therefore overestimate real-world performance compared with the more challenging cross-docking scenario, where both receptor and ligand conformations differ from the experimental reference. Second, despite the emergence of Boltz-2 as a next-generation biomolecular structure-prediction framework, no peer-reviewed study has directly compared AlphaFold 3 and Boltz-2 using a standardised protein–ligand pose-prediction benchmark. As a result, their relative strengths, weaknesses, and generalisation capabilities remain poorly understood. Third, the influence of binding-site architecture on docking performance has not been systematically evaluated across modern physics-based, deep-learning, and diffusion-based methods, particularly regarding cavity depth, enclosure, conformational gating, and solvent accessibility.

Here, we address these gaps through a comprehensive benchmarking study of three pharmacologically important targets, AChE, BACE1, and GSK-3β, which span diverse binding environments ranging from deep, enclosed gorges to flap-regulated cavities and solvent-exposed pockets. Using standardised redocking and cross-docking protocols, we compare seven representative methods encompassing physics-based, deep-learning, and diffusion-based paradigms. By directly benchmarking AlphaFold 3 and Boltz-2, while systematically evaluating the effects of receptor conformation and binding-site architecture, we identify key determinants of docking success and provide practical guidance for selecting and integrating complementary computational approaches in structure-based drug discovery.

## Methodology

### Dataset extraction and preprocessing

74 AChE, 431 BACE1, and 107 GSK-3β protein–ligand complex structures were retrieved from the RCSB Protein Data Bank (PDB) (https://www.rcsb.org/). UCSF Chimera v1.16 (https://www.cgl.ucsf.edu/chimera/) was used to structurally align the structures for each target because of the different conformational diversity. Structures lacking co-crystallized small-molecule ligands were excluded from redocking and cross-docking analyses. To ensure docking consistency and prevent spurious interactions, all non-receptor entities were removed, including crystallographic solvents and ions (*e.g.*, water, sulphate, zinc, chloride, hydroxide, urea, phosphate, dimethyl sulfoxide, polyethylene glycol, tartaric acid, iodine, nickel, sodium, and glycerol). Only biologically relevant protein chains and associated ligands were retained for subsequent docking analyses.

### Protein and ligand preparation

**Fig. 1:**
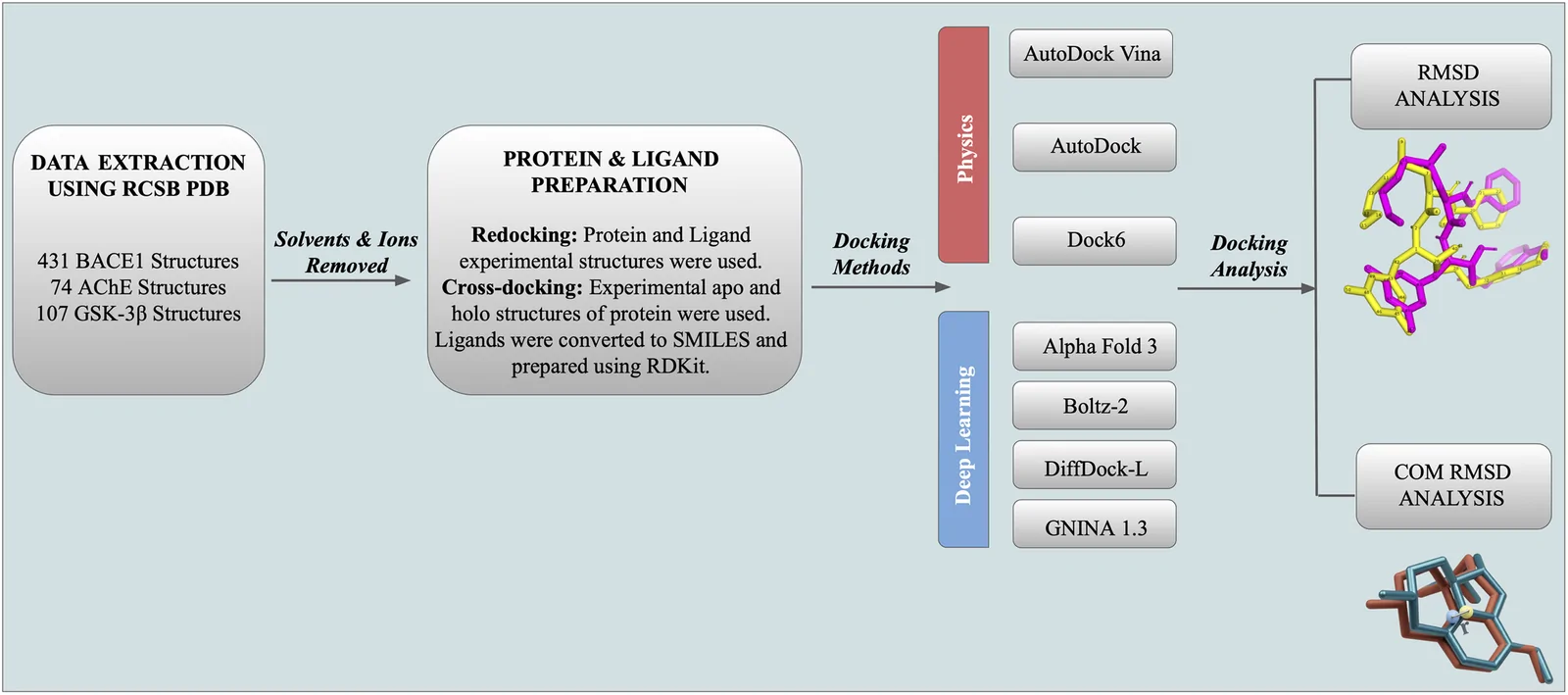
A comprehensive benchmarking workflow for evaluating physics-based and deep learning-based protein–ligand docking approaches. In the above figure, RMSD analysis refers to heavy-atom root mean square deviation, and COM RMSD analysis refers to the center-of-mass root mean square deviation.

Protein and ligand preparation were performed to ensure structural consistency across physics-based and DL-based docking methods, including AutoDock Vina, AutoDock4, GNINA 1.3, and DOCK 6. Two docking scenarios were considered: redocking and cross-docking. For cross-docking, we selected one holo and one apo receptor structure for each target based on two objective criteria: (i) highest available X-ray diffraction resolution, and (ii) fewest missing residues in the binding site region. This approach ensures optimal experimental reliability and reproducibility. The selected structures and associated resolution values were: AChE (holo: 4 M0E, 1.55 Å; apo: 5HF5, 2.30 Å), BACE1 (holo: 7MYI, 1.95 Å; apo: 3TPJ, 2.00 Å), and GSK-3β (holo: 1J1B, 2.20 Å; apo: 1I09, 2.30 Å) as shown in Fig. 1. While different template choices may yield minor quantitative variations, our use of consistent, quality-based selection criteria ensures that the relative performance trends across methods and targets are robust and generalizable.

Ligands for cross-docking were first converted to SMILES and subsequently regenerated using RDKit, thereby preserving the molecular connectivity and stereochemical assignments encoded in the input structures while eliminating dependence on the original crystallographic conformations. The regenerated ligand structures were then geometry-optimized and energy-minimized using Open Babel. For redocking, the experimentally resolved ligand coordinates were retained as the starting structures and placed back into their corresponding co-crystallized receptor structures. AutoDock4 and AutoDock Vina, ligands were prepared using MGLTools v1.5.7, including hydrogen addition, charge assignment, and geometry refinement. Protein structures were also prepared using MGLTools v1.5.7 by removing alternate atom conformations, adding polar hydrogens, assigning Gasteiger charges, and standardizing atom types. For DOCK 6, both ligands and receptor structures were prepared using UCSF Chimera v1.16 by adding hydrogens and assigning AM1-BCC charges to appropriately represent their molecular interaction properties. Thus, although the initial ligand preparation differed between redocking and cross-docking to minimise conformational bias, the resulting structures were processed using the standard preparation procedures required by each docking method.

No systematic enumeration of alternative protonation states or tautomers was performed. Instead, the chemical representation specified by the experimental structure was retained for redocking, whereas the chemical representation encoded by the corresponding input SMILES was retained for cross-docking throughout the benchmark. This approach ensured that differences in docking performance were not introduced by systematic variation in protonation or tautomeric states.

Before docking, ligands were additionally grouped according to structural similarity using RDKit Morgan fingerprints and Tanimoto similarity. The Butina algorithm was used to cluster structurally similar ligands into groups.

### Docking setup

Rigid and flexible docking calculations were performed using AutoDock Vina, GNINA 1.3, and AutoDock4. The docking grid box was centred on experimentally determined binding pocket coordinates: AChE (2.799, 0.0, 7.0), BACE1 (−7.0, 5.4, −6.2), and GSK-3β (−1.296, 9.007, −7.703). AutoDock Vina and GNINA 1.3 employed a cubic grid of 30 × 30 × 30 Å^3^, while AutoDock4 used a grid box of 80 × 80 × 80 points (with a grid spacing of 0.375 Å) to cover the binding pocket fully. For DOCK 6, receptor surfaces were generated, and binding pockets were identified using spheres within 10 Å of the ligand. A grid box of 8 Å per dimension was defined for docking, and the ligands were energy-minimised before the calculation. In flexible re-docking, we used ligands with experimental coordinates and a single receptor (4M0E: AChE, 7MYI: BACE1, and 1I09: GSK-3β) in the calculations to ensure consistent residue mapping. In the BACE1 structure (PDB: 4J0Y), residue numbering begins at position 57 and extends to 446, reflecting the truncated construct. This introduces ambiguity when defining equivalent flexible residues across multiple structures, particularly in functionally critical regions such as the flap and active site.

All the AI methods (AlphaFold 3, Boltz-2, GNINA 1.3, and DiffDock) clarification of Software Versions, Input Parameters, Ranking Criteria, and Reproducibility is provided in Table S1. Flexible docking with GNINA 1.3 was performed using the same receptor preparation protocol as AutoDock Vina and AutoDock4 to ensure methodological consistency. The identical flexible receptor file was used, containing the selected side-chain residues, corresponding to the rigid receptor file.

DiffDock performs blind docking, predicting ligand poses across the entire protein surface without prior knowledge of binding sites. In AlphaFold 3 and Boltz-2, which are also blind docking-based methods, SMILES representations of ligands and protein sequences were used directly for docking and pose prediction (Table S1). Consequently, only cross-docking could be evaluated for these two methods.

A maximum of six flexible residues per receptor except AChE, for which seven flexible residues were chosen for flexible re-docking and cross-docking using holo-crystal structures to reduce computational cost while keeping ligand-induced conformational characteristics. Using contact analysis and prior research, we identified residues that directly interacted with the co-crystallised ligand within a 5 Å threshold in VMD and selected them for side-chain flexibility analysis. TRP86, SER203, HIS447, GLU334, PHE295, TRP286 and TYR337 were considered flexible for AChE. The catalytic triad that hydrolyses acetylcholine is made up of SER203, HIS447, and GLU334. TRP86 helps the enzyme recognise the substrate at the choline-binding site. PHE295 is part of the acyl-binding pocket. TYR337 acts as a gating residue in the catalytic gorge. TRP286, a key residue of the Peripheral Anionic Site (PAS) located at the entrance of the catalytic gorge, plays a critical role in substrate guidance, ligand recognition, and regulation of ligand access to the active site. For GSK-3β, flexible residues included LYS85, GLN185, ASN186, ASP200, ASP133, and TYR134. LYS85 participates in conserved salt-bridge formation and ATP inhibitor interactions. GLN185 and ASN186 form the hinge region critical for hydrogen bonding with ATP-competitive inhibitors. ASP200 belongs to the conserved DFG motif that governs kinase conformational switching, while ASP133 and TYR134 contribute to stabilising interactions near the ATP-binding site. Residue identities and numbering were verified by sequence and structural alignment prior to receptor preparation to ensure consistent definition of the flexible residues across all targets (Note S1 & Fig. S1). For BACE1, side-chain flexibility was allowed for ASP32, ASP228, TYR71, TRP76, ARG128, and THR231. ASP32 and ASP228 form the catalytic dyad. The flap-region residue, TYR71, regulates entry to the active site. THR231 helps place the ligand using hydrogen-bond networks, ARG128 provides electrostatic stabilisation, and TRP76 adds hydrophobic and π-stacking interactions.

### Heavy atom RMSD and COM RMSD analysis

Heavy atom root-mean-square deviation (HA-RMSD) (or will be simply referred to as RMSD) and centre of mass (COM) RMSD were used to evaluate docking accuracy. HA-RMSD is a common metric for measuring structural divergence between predicted and observed ligand binding positions and was used to evaluate docking accuracy. Only heavy atoms were included in the HA-RMSD calculations, as hydrogen atom positions are often not experimentally resolved and are typically added computationally during structure preparation. Inclusion of hydrogen atoms can therefore introduce methodological variability unrelated to docking performance, potentially biasing. HA-RMSD measures positional differences between corresponding ligand atoms, with lower values indicating closer agreement and higher docking accuracy. For a ligand with P atoms, HA-RMSD was calculated as the square root of the average squared Euclidean distance between experimental coordinates *x*_p_ and predicted coordinates *y*_p_ for each atom P. In addition, COM RMSD calculations were done to check global translational alignment and find overall positional shifts of ligands within the binding pocket. The COM RMSD was calculated using the mass-weighted average of heavy atoms only, where each atomic position *r*_p_ was weighted by its atomic mass *w*_p_ and normalised by the total molecular mass.

Docking performance was benchmarked using an HA-RMSD cutoff of 2 Å relative to the crystallographic reference pose. HA-RMSD and COM RMSD deviations were grouped into 2 Å bins (≤2 Å, >2–4 Å, *etc.*) and computed for ten poses generated per docking run. Performance was evaluated using two complementary criteria: the minimum HA-RMSD pose, which shows how well a method can find conformations close to the native state (the predicted binding poses with the least HA-RMSD compared to the experimental binding pose), and the best-scoring pose, defined as the pose with the most favorable predicted binding affinity (the lowest docking energy), whose HA-RMSD assesses how well the scoring function ranks native-like solutions. For AlphaFold 3, Boltz-2, and DiffDock, which do not provide directly comparable conventional docking-energy scores, the generated or ranked top pose was considered the best pose for consistency of evaluation.
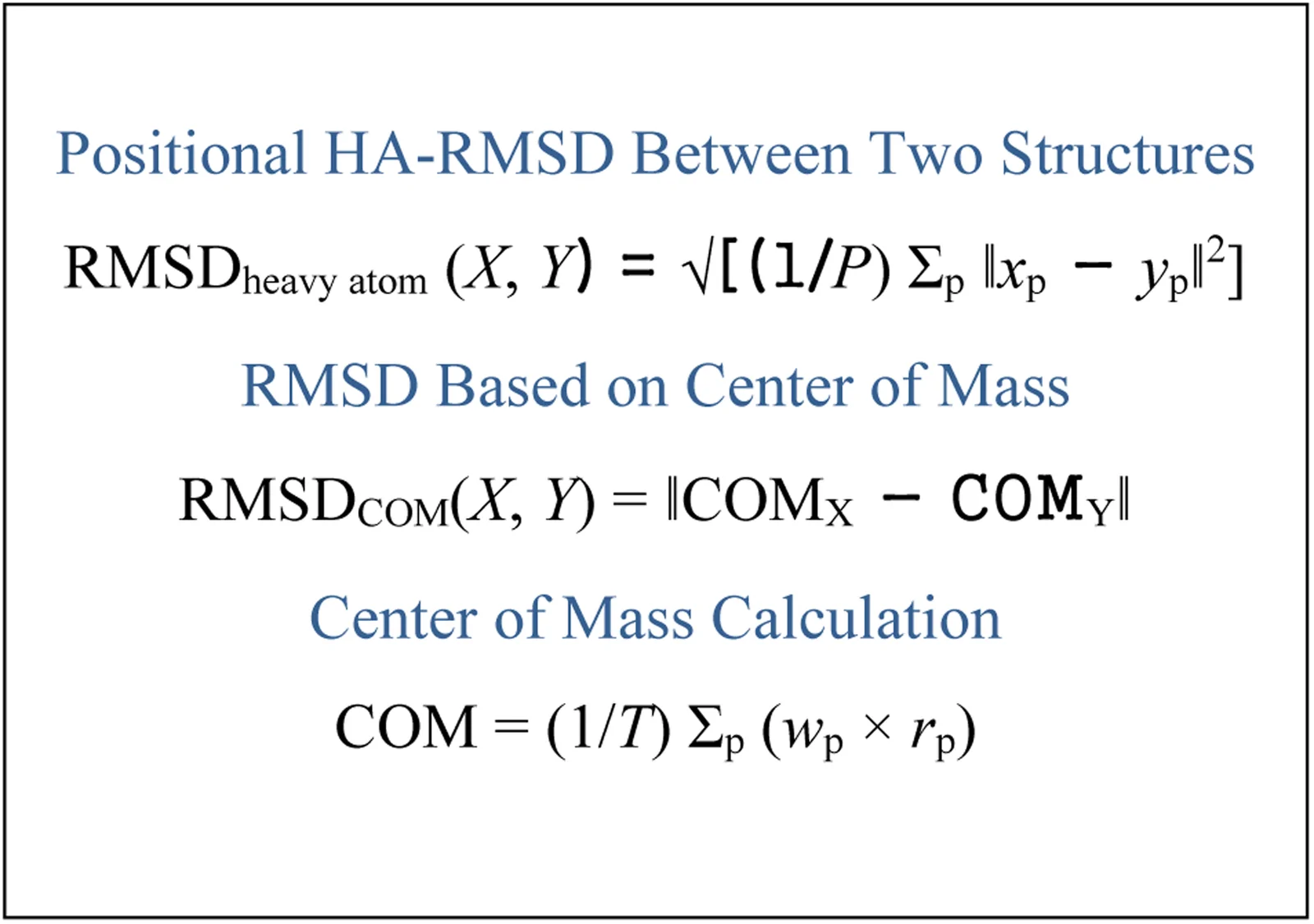


### Vacant volume calculations

Vacant pocket volumes were calculated using LigBuilder V3. Cavity detection was performed in whole-protein mode using the default cavity detection parameters and default cavity settings. The default hybrid surface/volume detection algorithm was used without modification. The same cavity detection protocol and parameter settings were applied to all experimental structures and representative flexible-docking conformations, ensuring that vacant pocket volumes were calculated consistently and were directly comparable across all analyzed structures. All predicted cavities were visually inspected, and the cavity corresponding to the experimentally observed ligand-binding site was selected for subsequent vacant pocket volume analysis.

### Statistical analysis for all docking tools

Docking performance was evaluated for each method. HA-RMSD values were summarised as mean ± SD and median (IQR), with docking success defined as HA-RMSD ≤ 2.0 Å. Success rates were reported with two-sided 95% Wilson confidence intervals. Pairwise comparisons between methods were performed using only matched protein–ligand complexes with valid predictions from both methods. HA-RMSD distributions were compared using the paired Wilcoxon signed-rank test, whereas differences in docking success rates were assessed using McNemar's test. *P*-values were adjusted for multiple comparisons using the Holm correction, with adjusted *p*-values < 0.05 considered statistically significant. All statistical tests were two-sided.

### Understanding memorization in deep learning methods

To assess the performance of the evaluated DL-based docking methods on complexes that may have been encountered during model development, we systematically quantified the presence of the benchmark complexes in the reported training datasets. This analysis was used to evaluate whether the methods retained accurate docking performance for complexes with documented training-data exposure. The overlap analysis was performed for the crossdock results of GNINA 1.3, DiffDock, AlphaFold 3, and Boltz-2, and was also assessed for the redock results of GNINA 1.3 and DiffDock.

### Statistical analysis of training-data overlap

Prediction performance was compared between paired complexes present in training-data using Wilcoxon signed-rank tests for continuous HA-RMSD values and McNemar's tests for paired success/failure classifications, where applicable. Comparisons were restricted to complexes for which both conditions yielded valid predictions, and *P* values were adjusted for multiple comparisons using the Holm correction. Cross-method comparisons were primarily performed under the shared crossdock condition to maintain a common evaluation setting.

## Results

Docking accuracy was evaluated using redocking and cross-docking approaches. Redocking assesses a method's ability to reproduce the native ligand pose within its co-crystallised receptor, providing an estimate of intrinsic accuracy under ideal conditions. In contrast, cross-docking evaluates performance when ligands are transferred to different receptor conformations (apo and holo), in which both receptor structures and ligand coordinates differ from those of the original experimental complex, thereby capturing receptor flexibility and conformational variability across targets. Both rigid and flexible docking protocols were employed with AutoDock4, AutoDock Vina, and GNINA 1.3 to assess how receptor adaptability affects docking accuracy. In rigid docking, sampling is limited to ligand translational, rotational, and torsional degrees of freedom while keeping the receptor conformation fixed, which approximates a physics-based lock-and-key model with high computational efficiency. Flexible docking allows specific binding-site residues to sample different conformations, thereby expanding this framework and partially capturing induced-fit effects, but it also complicates the search space.

Before docking, cluster analysis was employed to assess ligand diversity and reduce redundancy-driven bias. For BACE1, 296 clusters out of 415 ligands were discovered; AChE generated 36 clusters from 54 ligands, and GSK-3β generated 86 clusters from 106 ligands, indicating a high level of chemical diversity. After the identical ligands were removed, the final datasets contained 20 ligands for AChE, 69 ligands for GSK-3β, and all 415 ligands for BACE1. This preprocessing ensured that performance measurements appropriately reflected methodological robustness rather than over-representing closely related chemotypes.

### Cross-docking analysis and the effect of receptor conformation on docking accuracy

**Fig. 2:**
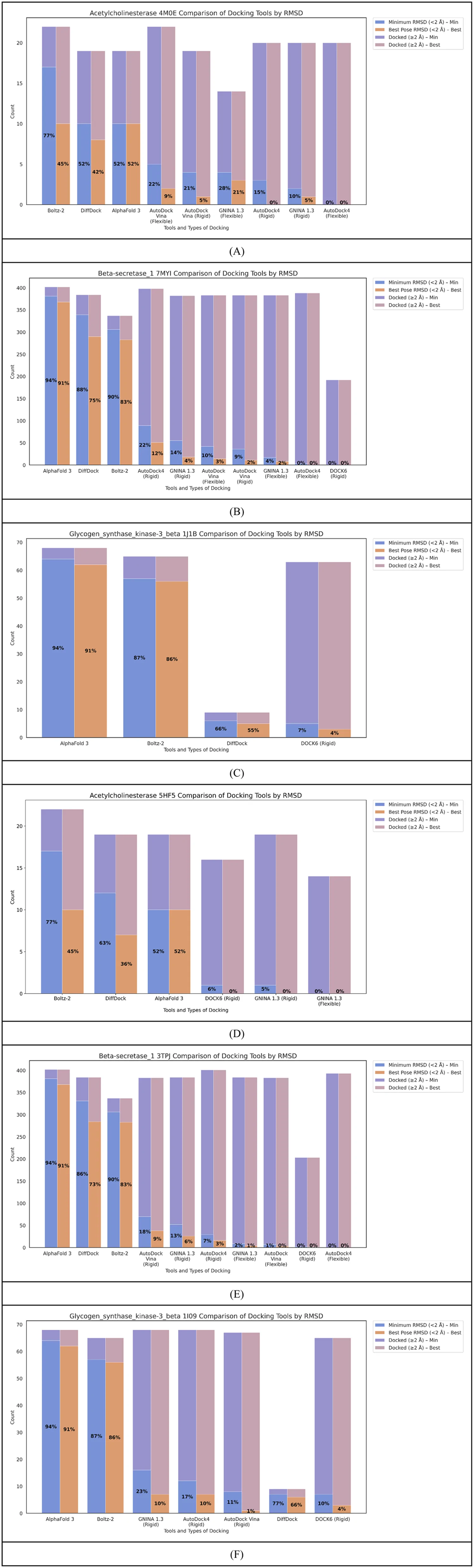
Cross-docking-based HA-RMSD analyses for the holo and apo forms of AChE, BACE1, and GSK-3β. Panels A, B, and C show HA-RMSD-based analyses for the holo receptor, whereas panels D, E, and F show HA-RMSD analyses for the apo form. The *y*-axis represents the success rate (%), defined as the percentage of docking runs with HA-RMSD ≤ 2 Å. DL-based approaches such as Boltz-2 and AlphaFold 3 bypass the conventional apo–holo paradigm altogether. In panel C, only four tools exhibited HA-RMSD values greater than 0%; therefore, only these are shown.

**Fig. 3:**
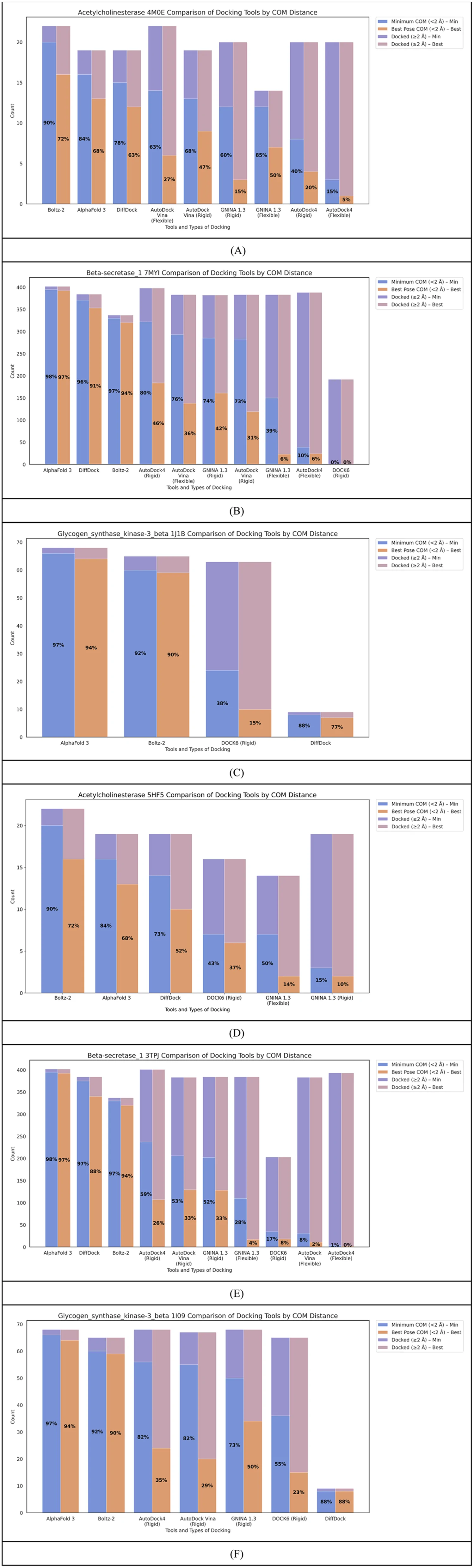
Cross-docking-based COM RMSD analyses for the holo and apo forms of AChE, BACE1, and GSK-3β. Panels A, B, and C show COM-RMSD-based analyses for holo form and panels D, E, and F show COM-RMSD analyses for apo form that quantify the spatial overlap between docked ligands and experimental ligand positions. DL-based approaches such as Boltz-2 and AlphaFold 3 yield bypassing the conventional apo–holo paradigm altogether, as the FASTA sequences of target proteins are used as inputs.

The conformational state of the receptor significantly affects docking performance across all three targets. Holo structures consistently produced greater pose recovery for AChE and BACE1 than apo forms, whereas GSK-3β performed better in the apo state (Fig. 2 and 3). Ligand binding helps AChE recognise ligands better by keeping important side-chain orientations in the catalytic gorge and peripheral anionic site.^16^ Ligand binding to BACE1 enables precise accommodation of substrates and inhibitors by stabilising the catalytic aspartates and organising the flexible flap region.^17^ Such conformational ordering improves docking accuracy by limiting conformational sampling and reducing steric conflicts.^18^ However, DL-based techniques that do not rely on the apo–holo paradigm, such as AlphaFold 3 and Boltz-2, directly predict protein–ligand complexes from sequence and SMILES representations.

To determine whether the observed differences in docking performance could be explained by intrinsic structural features of the binding sites, we next analysed the geometry and topology of the receptor cavities. Because ligand accommodation is strongly influenced by pocket size, depth, enclosure, and solvent accessibility, cavity analysis can provide a mechanistic framework for interpreting target-specific differences in pose recovery and the contrasting behaviour of apo and holo structures.

LigBuilder V3 was used for binding-site cavity analysis to identify the architectural basis of the binding site for all three targets. Fig. 5 shows the intrinsic variations in cavity architectural depth, enclosure, and solvent exposure among the three targets. For BACE1, the apo form (3TPJ) is more compact (with volume and surface area, respectively as 1975.63 Å^3^ and, 1100.00 Å^2^), whereas the holo structure (7MYI) exhibits a larger cavity (2334.63 Å^3^; 1332.25 Å^2^), reflecting differences in active-site geometry. The partially enclosed cavity geometry observed in Fig. 5B supports tighter ligand confinement and improved pose discrimination. The sequence alignment was performed to verify residue-numbering correspondence between BACE1 apo (PDB: 7MYI) and holo (PDB: 3TPJ) structures prior to flexible docking. The alignment confirmed the correspondence of the catalytic residues (Asp32 and Asp228) and flap residue (Tyr71), ensuring correct flexible-receptor definition. The detailed sequence alignment and residue correspondence are provided in Note S1 and Fig. S1. In AChE, the apo form (5HF5) expands to 1224.75 Å^3^, whereas the holo structure (4M0E) has a smaller but highly complex cavity (941.75 Å^3^). Its structural intricacy is underpinned by a deep gorge with several subpockets that facilitate selective ligand recognition (Fig. 5A). In contrast, the inhibitor-bound GSK-3β structure (PDB: 1J1B) possesses a smaller binding cavity (1482.00 Å^3^), whereas the more open structure (PDB: 1I09) exhibits an enlarged cavity (1886.63 Å^3^). Structural alignment of the activation loop (residues 200–226) revealed a backbone HA-RMSD of only 0.26 Å, indicating that the increased cavity volume arises from subtle local conformational adjustments rather than large-scale loop rearrangements. The enlarged, solvent-exposed pocket reduces geometric constraints on ligand placement, increasing the number of energetically similar binding orientations (Note S2, Fig. S2 & Table S3).

The contrasting behaviour of the three targets suggests that receptor cavity architecture, rather than receptor state alone, is a primary determinant of docking success. For AChE and BACE1, ligand binding promotes a more pre-organised binding environment, facilitating pose recovery. In contrast, the apo GSK-3β structure exhibits an enlarged, more solvent-exposed ATP-binding pocket that provides greater accessibility to the binding site, resulting in improved docking performance relative to the inhibitor-bound structure. These findings indicate that holo structures are not universally optimal for docking studies. Instead, the choice between apo and holo receptor conformations should be guided by the structural consequences of ligand binding on pocket geometry. Binding sites that become more enclosed upon ligand binding are likely to benefit from holo templates. In contrast, targets exhibiting ligand-induced pocket expansion may achieve superior docking performance using apo conformations.

DL-based methods outperformed physics-based docking approaches in pose recovery across all three targets. AlphaFold 3 and Boltz-2 consistently achieved the highest pose-recovery accuracy among DL approaches, based on the minimum HA-RMSD criterion, followed by DiffDock, demonstrating that end-to-end learning-based structural prediction frameworks are more resilient than conventional scoring-based docking. AlphaFold 3 outperformed Boltz-2 in all three scenarios when considering the best binding pose. Interestingly, despite the under-representation of AChE and GSK-3β in the AlphaFold 3 and Boltz-2 training datasets, both models exhibited strong generalisation, with minimum HA-RMSD pose recovery accuracies of approximately 52% and 77% for AChE, and 94% and 87% for GSK-3β, respectively. This indicates that both models can generalise beyond previously encountered structures, potentially by learning fundamental physicochemical and geometric principles governing protein–ligand interactions. However, compared with GSK-3β, the relatively limited structural representation of AChE appears to have negatively affected prediction performance, highlighting the importance of training data diversity and coverage for DL-based models, particularly for targets exhibiting substantial conformational flexibility or structural diversity.

For AChE, AlphaFold 3 was trained on fewer experimental structures (67) than Boltz-2 (74). Consequently, Boltz-2 achieved slightly better performance when the lowest HA-RMSD pose was considered, whereas AlphaFold 3 consistently produced more accurate top-ranked predictions. This observation highlights the importance of confidence calibration in generative protein–ligand models. Generating a near-native binding pose is only one component of successful pose prediction; the model must also reliably identify and rank that pose above less accurate alternatives. The superior performance of AlphaFold 3 for top-ranked predictions suggests that its confidence estimation is better aligned with structural accuracy, enabling more reliable pose selection despite comparable sampling capability. This distinction is particularly important for practical virtual screening workflows, where only the highest-ranked prediction is typically subjected to downstream analysis or experimental validation. These findings therefore indicate that improvements in confidence calibration and ranking strategies may be as important as improvements in conformational sampling for future AI-based protein–ligand prediction methods.

In the cross-docking evaluation, the highest-performing methods varied across targets and between holo and apo structures. For holo AChE, DiffDock (with 52% success rate) and AutoDock Vina (rigid) (21%) performed best. For holo BACE1, DiffDock (88%) and AutoDock4 (rigid) (22%) showed the highest performance, while for holo GSK-3β, DiffDock (66%) and DOCK 6 (7%) performed best (Fig. 2 and Table S2). For apo structures, DiffDock performed best for AChE (63%), followed by DOCK 6 (6%). For BACE1, DiffDock achieved the highest performance (86%), followed by AutoDock Vina (18%). For GSK-3β, DiffDock (77%) and GNINA 1.3 (rigid) (23%) showed the highest performance. COM RMSD analysis further assessed the consistency of docking performance across the different structures and docking protocols. For holo AChE, DiffDock, AutoDock Vina, and GNINA 1.3 achieved COM RMSD accuracies exceeding 60%. For holo BACE1, DiffDock, AutoDock Vina, GNINA 1.3 (rigid), and AutoDock4 (rigid) each achieved COM RMSD accuracies above 73%. In contrast, none of the evaluated methods achieved substantial COM RMSD accuracy for holo GSK-3β. For apo AChE, GNINA 1.3 (flexible) and DiffDock achieved COM RMSD accuracies above 50%, whereas for apo BACE1, AutoDock Vina (rigid) and rigid AutoDock4 exceeded 50% COM RMSD accuracy. For apo GSK-3β, DOCK 6, AutoDock Vina (rigid), AutoDock4 (rigid), and GNINA 1.3 (rigid and flexible) demonstrated COM RMSD accuracies above 50% (Table S2).

Correct pocket localisation and correct binding-pose recovery were evaluated using complementary metrics. COM RMSD assesses whether the predicted ligand is placed within the correct binding pocket. In contrast, HA-RMSD evaluates the accuracy of the ligand's atomic orientation and conformation relative to the experimental structure. Thus, cases with low COM RMSD but high HA-RMSD were interpreted as successful pocket localisation but unsuccessful pose recovery. These representative complexes were manually inspected using UCSF Chimera, confirming that the ligand occupied the correct binding site despite adopting an incorrect orientation or conformation within the pocket (Fig. S3).

### Evaluation of CNN-based and empirical scoring functions for cross-docked pose selection using the 7MYI holo receptor

GNINA 1.3 provides three scoring configurations that enable separation of the effects of empirical scoring, CNN rescoring, and CNN-guided refinement. To determine whether the 3D CNN scoring function is more susceptible than the empirical Vina scoring function to receptor perturbations, we performed a comparative analysis using the 7MYI holo receptor with cross-docked ligands. Three GNINA 1.3 configurations were evaluated: (i) empirical Vina scoring only, (ii) CNN rescoring, and (iii) CNN rescoring with refinement. For each configuration, we compared the best pose success rate, representing the pose selected as top-ranked by the scoring function, with the minimum HA-RMSD success rate, representing the lowest HA-RMSD pose sampled regardless of its rank.

CNN rescoring and CNN rescoring with refinement progressively improved docking performance relative to empirical Vina scoring alone. The best pose success rate increased from 2% for empirical scoring to 4% with CNN rescoring and 9% with CNN refinement, while the minimum HA-RMSD success rate increased from 7% to 11% and 15%, respectively (Fig. S4). These findings demonstrate that CNN-based refinement generates a higher proportion of near-native candidate poses than empirical scoring alone.

However, the difference between the best pose and minimum HA-RMSD success rates remained relatively constant across all three configurations (approximately 5–7 percentage points). Since this gap reflects the scoring function's ability to identify the best-sampled pose correctly, the results indicate that although CNN-guided refinement improves pose generation, it provides only limited improvement in ranking accuracy relative to the empirical Vina scoring function. Under the receptor perturbations introduced by cross-docking, the principal limitation therefore appears to be pose discrimination rather than conformational sampling. These observations suggest that both empirical and CNN-based scoring functions are similarly challenged by subtle conformational differences arising from receptor flexibility. At the same time, CNN-guided refinement primarily enhances the quality of the sampled pose ensemble rather than the selection of the optimal pose.

### DiffDock redocking and cross-docking analysis

**Fig. 4:**
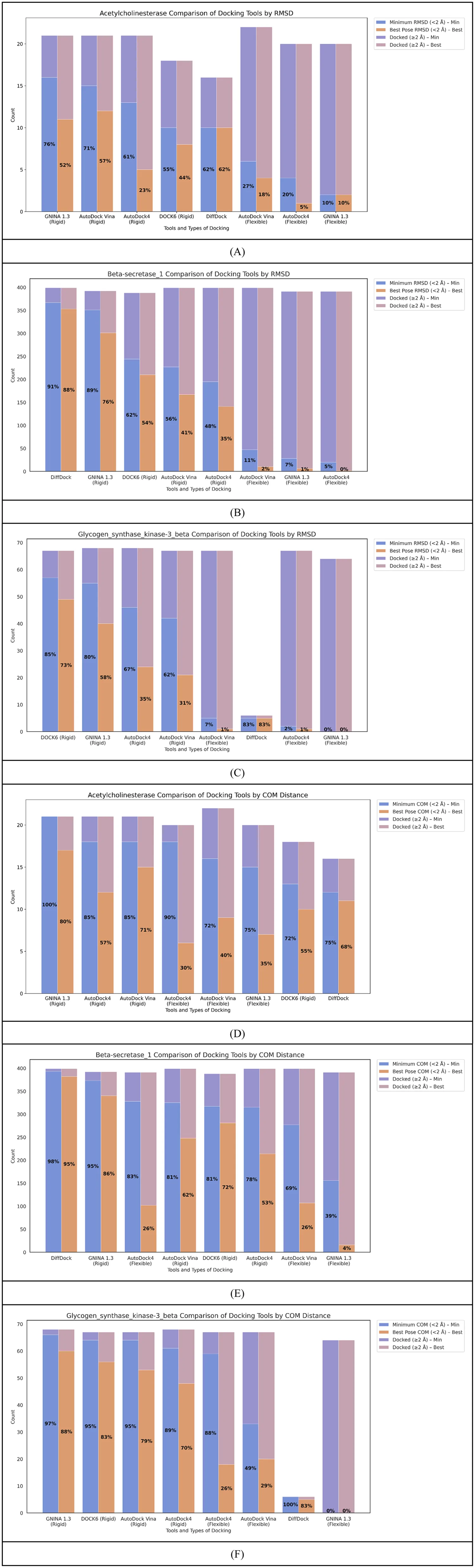
Redocking HA-RMSD & COM RMSD analysis of AChE, BACE1, and GSK-3β. Redocking-based HA-RMSD and COM RMSD analysis for AChE, BACE1, and GSK-3β. Panels A, B, and C show HA-RMSD-based analyses of docked ligands, which reflect how much the predicted ligand poses differ from their crystallographic reference structures. Panels D, E, and F show COM RMSD analyses that quantify the spatial overlap between docked ligands and experimental ligand positions. The *y*-axis represents the success rate (%), defined as the percentage of docking runs with HA-RMSD or COM RMSD ≤ 2 Å.

Across all three targets, DiffDock achieved higher pose prediction accuracy under redocking than under cross-docking, with the difference most pronounced for Top-1 predictions. This advantage was substantially reduced when the best of ten predicted poses was considered. For GSK-3β, the best pose success rate was 83% under redocking, compared with 66% (holo) and 77% (apo) under cross-docking. For BACE1, success was 91% under redocking *versus* 88% (holo) and 86% (apo) and for AChE, success was 62% under redocking, compared with 52% (holo) and 63% (apo) (Fig. 2–4). For three of the targets, apo form performed best in cross-docking with DiffDock.

**Fig. 5:**
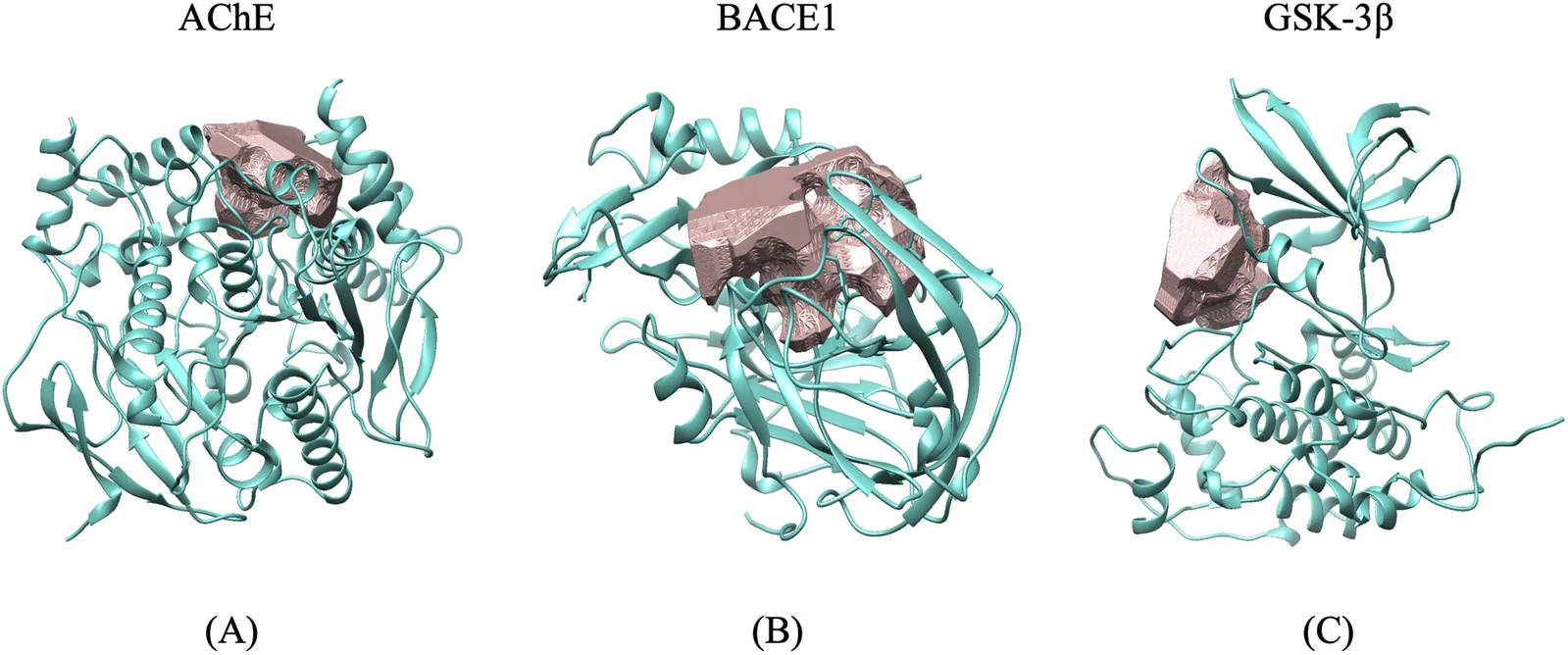
Structural comparison of binding-site cavities of AChE, BACE1, and GSK-3β. Protein structures are shown as cyan cartoons, with binding-site cavities rendered as semi-transparent pink surfaces. (A) AChE features a deep, narrow gorge with multiple interconnected subpockets. (B) BACE1 exhibits a centrally located cavity beneath the flap region, forming a partially enclosed binding environment. (C) GSK-3β displays a comparatively open and solvent-exposed pocket with limited enclosure. These structural differences in cavity depth, topology, and accessibility influence ligand accommodation and docking behaviour.

DiffDock accepts ligand structures in several formats, including SMILES, SDF, and MOL2.^10^ During model development, ligand three-dimensional conformations were generated from SMILES using RDKit's ETKDG protocol, providing standardised initial geometries. In our workflow, however, the performance of the resulting ligand structures depended on the method used for structure preparation. For both cross-docking and redocking ligands, adding hydrogens using RDKit did not consistently yield correct ligand structures. Similarly, structures prepared by adding hydrogens using UCSF Chimera also did not show comparable redocking accuracy. In contrast, ligand conversion and subsequent structure processing for both cross-docking and redocking were performed separately using Open Babel; these structures were therefore used as SDF inputs.

To assess whether sensitivity to the initial ligand conformer contributed to the observed performance differences, we evaluated DiffDock's robustness to stochastic ETKDG initialisation. We selected 97 BACE1 ligands containing ≥10 rotatable bonds, as highly flexible compounds were expected to be most sensitive to differences in starting geometry. For each ligand, ten independent conformers were generated using different ETKDG random seeds and docked independently with DiffDock. The resulting Top-1 pose HA-RMSDs were evaluated against the corresponding experimental crystal structures.

As shown in Fig. S5, most ligands exhibited limited variation in Top-1 HA-RMSD across the ten conformer seeds, with within-ligand standard deviations generally below approximately 1.5 Å. A small subset showed substantially greater variability, with standard deviations exceeding 8 Å and reaching approximately 9–12 Å in the most variable cases. Success/failure classification changes were concentrated primarily among ligands with HA-RMSDs near the 2 Å threshold. In contrast, ligands clearly above or below the threshold generally remained consistently classified across seeds. Across the dataset, the median within-ligand standard deviation was 0.174 Å (mean, 0.469 Å), and 19 of 97 ligands (19.6%) changed success/failure classification across conformer seeds. These findings indicate that stochastic ETKDG initialisation has little effect on most ligands, although a minority of highly flexible compounds remain sensitive to the starting geometry.

Taken together, these results suggest that the lower cross-docking performance is unlikely to be driven primarily by stochastic variation in ETKDG-generated conformers. Rather, it more likely reflects the additional structural challenge of predicting ligand poses in an alternative receptor conformation, as well as potential differences between experimentally observed bound-ligand geometries and the standardised conformers used during model development. This interpretation is consistent with recent work showing that DiffDock performs competitively as a pose-sampling method in cross-docking and virtual-screening benchmarks. Nevertheless, ETKDG-based conformer generation represents a potential limitation for conformationally strained ring systems and macrocycles, where knowledge-based torsion preferences may not adequately sample macrocycle conformations. This could, in turn, influence the apparent pose-prediction accuracy for such chemotypes and should be considered when interpreting the sensitivity analysis. At the same time, DiffDock can achieve improved virtual-screening performance when its generated poses are combined with established scoring functions such as AutoDock Vina, GNINA 1.3, or RTMScore.^19^

Overall, these results indicate that redocking provides a more favourable assessment of DiffDock's best pose prediction capability. In contrast, cross-docking provides a more stringent test of pose prediction when the receptor conformation differs from that of the experimentally determined complex. The substantially smaller differences observed for best-of-ten predictions suggest that considering multiple generated poses can reduce the apparent performance gap between redocking and cross-docking, indicating that near-native poses may still be sampled even when they are not ranked first. This distinction is particularly relevant to prospective virtual screening, where the receptor structure available for docking may not correspond to the precise binding-site conformation adopted by a ligand. Thus, cross-docking may provide a more realistic assessment of the robustness of pose prediction under receptor–structure uncertainty.

### Redocking analysis

Redocking results confirmed that rigid docking protocols are generally consistent and reliable across all three targets. GNINA 1.3 (rigid) and AutoDock Vina achieved >50% correct pose recovery for AChE based on both minimum HA-RMSD and best-scoring poses (Fig. 4 and Table S5), indicating a strong correspondence between scoring functions and geometric accuracy. For BACE1, GNINA 1.3 (rigid) and DOCK 6 exceeded the 50% threshold under both pose selection criteria, and a similar trend was observed for GSK-3β (Fig. 4 and Table S5).

GNINA 1.3 (rigid) demonstrated the most robust overall performance when COM RMSD accuracy was considered, achieving >50% recovery for both minimum HA-RMSD and best-scoring poses across all targets (Fig. 4 and Table S2). In comparison, AutoDock Vina reached this threshold for AChE, whereas GNINA 1.3 (rigid) satisfied it for BACE1 and GSK-3β. Collectively, these results suggest that GNINA 1.3 provides superior spatial discrimination, likely due to its convolutional neural network-based scoring function, which captures local protein–ligand interaction patterns more effectively than conventional physics-based approaches.

Flexible docking protocols exhibited greater variability and generally lower pose-recovery accuracy than rigid docking across all targets (Fig. 2 and 4). Despite this, COM RMSD based analysis indicates that flexible docking poses are frequently positioned within the correct binding site, suggesting that errors arise primarily from pose orientation and ranking rather than site identification (Fig. 4). Among flexible approaches, AutoDock (flexible) performed best, achieving ≥69% success rate for minimum COM RMSD poses and outperforming both AutoDock Vina and GNINA 1.3 under comparable conditions (Fig. 4).

Overall, these findings indicate that incorporating receptor flexibility does not necessarily improve pose ranking in well-defined binding pockets like BACE1 or GSK-3β. Instead, it expands the conformational search space without a proportional gain in sampling efficiency, thereby reducing predictive accuracy (Fig. 2 and 4). Current flexible docking implementations typically restrict side-chain motion to a limited subset of binding-site residues, which may further limit their ability to capture experimentally observed pocket heterogeneity.

**Fig. 6:**
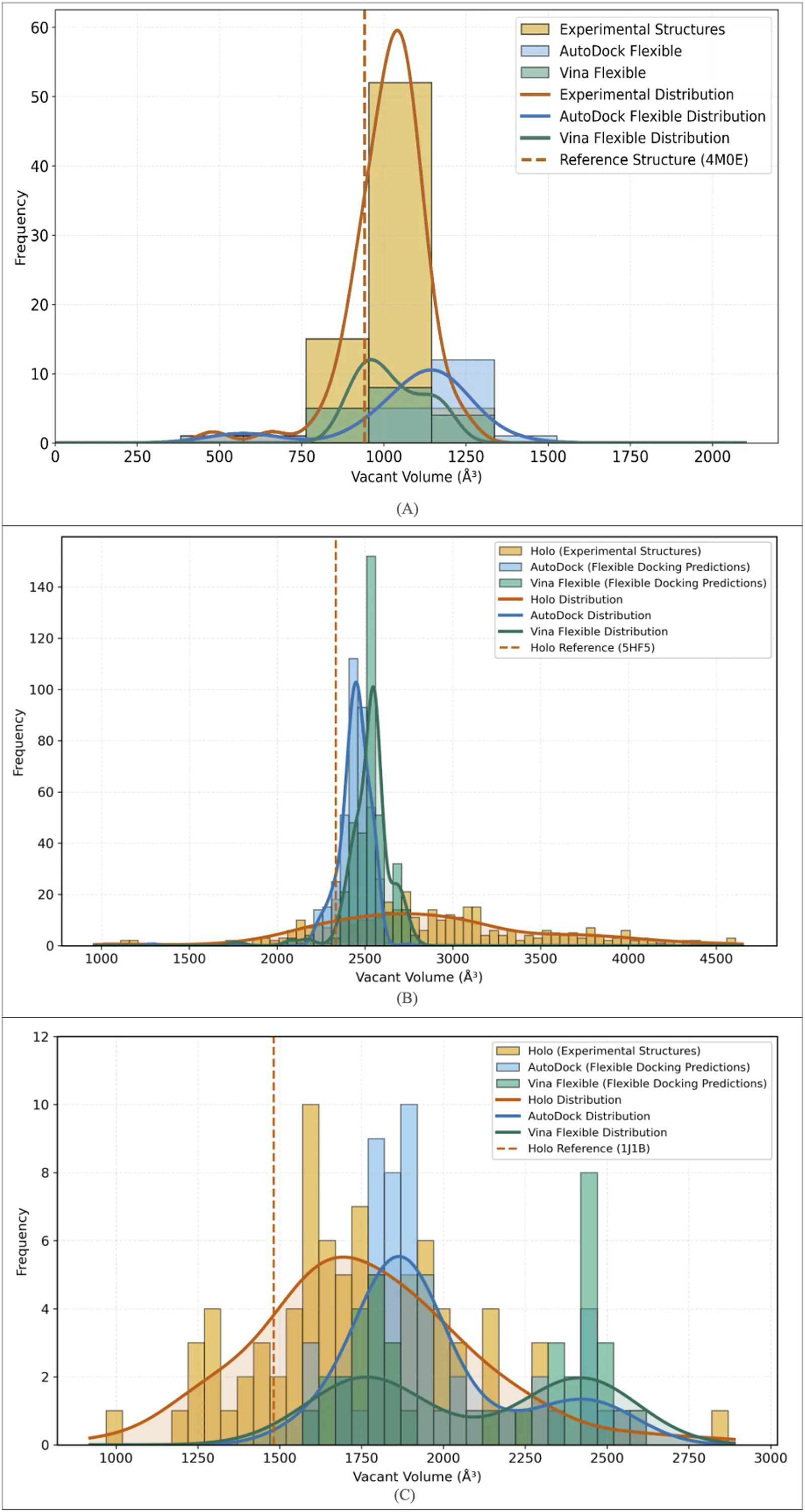
Distribution of vacant binding pocket volumes across experimental holo structures and flexible docking predictions (AutoDock & Vina) for (A) AChE, (B) BACE1 and (C) GSK-3β.

To evaluate this limitation, binding-pocket volumes derived from flexible docking were compared with those from experimental holo structures. As shown in Fig. 6, flexible docking produces narrowly distributed, approximately Gaussian pocket-volume profiles, whereas holo structures display broader distributions, reflecting greater conformational variability (Fig. 6). This discrepancy suggests that restricted side-chain sampling stabilizes a single dominant pocket state, thereby underrepresenting the intrinsic flexibility of the binding site.

In the present study, only a limited set of binding-site residues was treated as flexible to maintain computational feasibility across the large benchmarking dataset. Consequently, backbone motions and more extensive side-chain rearrangements were not sampled, restricting the accessible conformational space during docking. This limited flexibility likely contributed to the narrow pocket-volume distributions and the inferior performance of flexible docking relative to rigid docking, particularly for targets whose ligand recognition depends on larger-scale conformational changes.

We also observed that AutoDock was more prone to failures as ligand torsional flexibility increased, consistent with limitations noted in the AutoDock documentation for highly flexible ligands. AutoDock documentation also cautions that flexible-receptor calculations can produce unusually high binding energies when unfavourable internal interactions are introduced. In our results, although the docked ligands were positioned in the correct binding pocket, anomalous re-docking cases were dominated by the internal moving-fixed receptor term, whereas in cross-docking, both the receptor and ligand internal energy terms contributed to the inflated total energy. Importantly, the same inputs did not produce comparable anomalous energies in AutoDock Vina, supporting the interpretation that these extreme energies were artefacts of AutoDock's flexible-receptor sampling rather than genuine failures to identify the binding site.

### Ligand-size-normalized COM analysis for pose quality assessment

To refine the COM RMSD based success criterion, we evaluated the relationship between HA-RMSD and COM RMSD across all docking poses. HA-RMSD and COM RMSD showed a strong positive correlation (Pearson *r* = 0.93; Spearman *ρ* = 0.92; *p* < 0.001) (Fig. S6), confirming that COM RMSD displacement is a reliable descriptor of overall ligand positional deviation. Since absolute COM displacement can be affected by ligand size, COM RMSD was normalized by the ligand radius of gyration (COM RMSD per Rg) to obtain a size-independent metric.

Poses associated with local torsional deviations (HA-RMSD > 2 Å and COM RMSD < 2 Å) exhibited significantly lower COM RMSD per Rg values compared with poses showing global binding-site displacement (Mann–Whitney *U* test, *p* < 10^−16^). The 95th percentile of the torsional-deviation distribution corresponded to a COM RMSD per Rg value of 0.52, which was therefore adopted as an empirical, ligand-size-normalized threshold for distinguishing local torsional rearrangements from true binding-site misplacements. This analysis demonstrates that the normalized COM RMSD based criterion provides a more robust evaluation framework than a fixed 2 Å COM RMSD cutoff (Fig. S7 and S8).

### Statistical validation of docking and structure-prediction performance

Statistical rigour and generalisation performance were assessed across all evaluated tools using paired nonparametric analyses of matched protein–ligand complexes. A docking prediction was considered successful if its HA-RMSD from the native pose was ≤2.0 Å. Failed runs, including non-convergent executions and execution errors, were excluded from the success-rate denominator and reported separately as a measure of computational robustness. Success-rate confidence intervals were calculated using the Wilson score method. For paired comparisons, Wilcoxon signed-rank tests were used to assess differences in continuous HA-RMSD values, while McNemar's tests were used to compare binary success/failure outcomes on matched complexes. Holm–Bonferroni correction was applied across all pairwise comparisons to control the family-wise error rate (Tables S5 and S7). In redocking, GNINA 1.3 (Rigid) showed the strongest performance among the physics-based docking methods, achieving a success rate of 73.2% (95% CI, 69.0–76.9%) under minimum-energy pose selection and 87.7% (95% CI, 84.5–90.4%) when the minimum HA-RMSD pose was considered. GNINA 1.3 (Rigid) significantly outperformed the other physics-based methods in nearly all pairwise comparisons after Holm correction (adjusted *p* < 0.05; Table S7). However, this advantage did not translate to the more challenging cross-docking setting. Under crossdocking, its success rate fell sharply to 5.5% (95% CI, 4.2–7.2%). AutoDock Vina (Rigid) exhibited a similar decline, from 41.1% in redocking to 5.4% in crossdocking, reflecting a broader loss of performance across physics-based scoring methods when binding-site conformations differed from those used to generate the reference poses. DOCK 6 (Rigid) also showed a substantial increase in failed runs, rising from 4.3% in redocking to 43.5% in crossdocking, indicating a pronounced loss of computational robustness in addition to reduced docking accuracy. By contrast, learning-based structure-prediction methods maintained substantially higher performance under crossdocking. AlphaFold achieved a success rate of 90.0% (95% CI, 87.0–92.3%), followed by Boltz at 82.3% (95% CI, 78.4–85.6%) and DiffDock at 72.9% (95% CI, 69.8–75.9%). Each significantly outperformed all evaluated physics-based tools in crossdocking comparisons after Holm correction (adjusted *p* < 0.001; Table S5). Collectively, these results demonstrate a pronounced dependence of classical scoring-function-based docking methods on the availability of a binding-site conformation closely matched to the native state. Although such methods can achieve competitive or superior performance in redocking, their accuracy and, in some cases, computational robustness deteriorate substantially under crossdocking. Learning-based methods, in contrast, retain considerably higher success rates across alternative binding-site conformations, indicating stronger generalisation to the structural variability encountered in more realistic docking scenarios. Complete crossdocking and redocking summary statistics and pairwise significance tests are provided in Tables S4–S7 respectively.

### Evaluating structural memorization in deep learning models

Among the evaluated physics-based and deep learning (DL)-based methods, AlphaFold 3, Boltz-2, and DiffDock generally performed best under cross-docking conditions, while GNINA 1.3 and DiffDock achieved the highest accuracy in redocking. However, these results must be interpreted carefully because substantial overlap exists between the training datasets of several DL models and the benchmark complexes used for evaluation. Models trained on numerous PDB structures of frequently studied targets such as AChE, BACE1, and GSK-3β may therefore benefit from prior exposure to highly similar protein–ligand complexes. Consequently, excellent performance may partly reflect learned structural priors rather than complete generalization to previously unseen protein–ligand interactions.

AlphaFold 3 was trained using PDB entries released up to September 30, 2021,^8^ encompassing nearly all BACE1 structures and 19 of the 22 AChE complexes and 56 of the 69 GSK-3β complexes included in this study. DiffDock, trained on the PDBBind BLIND + dataset,^10^ and GNINA 1.3, trained using CrossDocked2020 v1.3 (ref. 11) (Table S1), likewise include substantial numbers of complexes related to these targets. Boltz-2 extends training to PDB entries released up to June 30, 2023,^9^ effectively covering nearly all benchmark complexes evaluated here. Such extensive overlap provides a stringent upper-bound scenario for assessing whether model performance primarily reflects memorization or genuine structural generalization.

Despite this extensive training-set coverage, pose-prediction accuracy remained far from perfect across all methods and targets. Failure was defined as a predicted ligand pose with HA-RMSD >2 Å from the crystallographic reference, the standard criterion for successful pose recovery. Considerable failure rates persisted even for complexes represented within the respective training datasets, demonstrating that prior exposure alone does not ensure accurate predictions.

Training-set overlap therefore does not guarantee accurate pose prediction. AChE provides a clear illustration of this limitation. Although nearly all benchmark structures were represented during training of AlphaFold 3, Boltz-2, and DiffDock, prediction failures remained common (Table 2). AlphaFold 3 and Boltz-2 exhibited substantially poorer performance (∼50%) for best pose. DiffDock showed only 20% failure in performance depending on the evaluation metric. Of the apo and holo structures, the receptor structure with the fewest prediction failures was included in the table below for DiffDock and GNINA 1.3. Importantly, AlphaFold 3 also accurately predicted several complexes absent from its training set (8DT7, 8AEN, and 8AEV), indicating genuine structural generalization beyond simple memorization.

**Table 2:** Number of protein–ligand complexes with HA-RMSD > 2 Å (failure rate) among systems present in the training data across different docking and structure prediction methods for AChE^a^

AChE (22)	Best_Pose_HA-RMSD	Best_Pose_COM_RMSD	Min_HA-RMSD_Pose	Min_COM_RMSD_Pose
Alpha fold 3 (16)	43.75%	25%	43.75%	6.25%
Boltz 2 (22)	54.54%	27%	22.72%	13.63%
DiffDock (cross-docking (holo)) (5)	20%	20%	0%	0%
DiffDock (re-docking) (4)	0%	0%	0%	0%
GNINA 1.3 rigid (cross-docking (holo)) (5)	100%	100%	80%	80%
GNINA 1.3 rigid (re-docking) (5)	0%	0%	0%	0%

a In column 1, the values represent the total number of docking results obtained from the different tools, relative to the number present in the training data.

A similar trend was observed for BACE1 (Table 3). Despite extensive representation of BACE1 complexes within the training datasets of AlphaFold 3, Boltz-2, and DiffDock, non-negligible failure rates persisted. AlphaFold 3 exhibited a best-pose failure rate of 8.5%, compared with 16.02% for Boltz-2, while DiffDock displayed substantial metric-dependent variability. Two BACE1 complexes (3KYR and 3EXO) represented particularly severe Boltz-2 failures, with all predicted poses exhibiting COM RMSD values exceeding 5 Å, consistent with major binding-site misplacement or difficulties associated with peptide-like ligands.

**Table 3:** Number of protein–ligand complexes with RMSD > 2 Å (failure rate) among systems present in the training data across different docking and structure prediction methods for BACE1^a^

BACE1 (415)	Best_Pose_HA-RMSD	Best_Pose_COM_RMSD	Min_HA-RMSD_Pose	Min_COM_RMSD_Pose
Alpha fold 3 (399)	8.52%	2.51%	5.26%	1.75%
Boltz 2 (337)	16.02%	5.04%	9.19%	2.37%
DiffDock (cross-docking (holo)) (320)	20.62%	6.56%	8.12%	2.5%
DiffDock (re-docking) (336)	8.93%	2.68%	5.36%	0.89%
GNINA 1.3 rigid (cross-docking (holo)) (78)	91%	62.82%	88.46%	43.58%
GNINA 1.3 rigid (re-docking) (79)	12.6%	6.3%	8.86%	6.3%

a In column 1, the values represent the total number of docking results obtained from the different tools, relative to the number present in the training data.

For GSK-3β, AlphaFold 3 again achieved the lowest overall failure rate (7.2%), whereas DiffDock and Boltz-2 exhibited greater variability and higher failure frequencies (Table 4). Notably, AlphaFold 3 successfully predicted 11 of the 13 complexes absent from its training dataset with HA-RMSD values below 2.5 Å, further demonstrating substantial generalization capability beyond previously observed structures.

**Table 4:** Number of protein–ligand complexes with RMSD > 2 Å (failure rate) among systems present in the training data across different docking and structure prediction methods for GSK-3β^a^

GSK-3β (69)	Best_Pose_HA-RMSD	Best_Pose_COM_RMSD	Min_HA-RMSD_Pose	Min_COM_RMSD_Pose
Alpha fold 3 (55)	7.2%	3.6%	7.2%	3.6%
Boltz 2 (64)	14.06%	9.38%	12.5%	7.81%
DiffDock (cross-docking (apo)) (6)	16.67%	0%	16.67%	0%
DiffDock (re-docking) (4)	25%	25%	25%	0%
GNINA 1.3 rigid (cross-docking (apo)) (42)	95.23%	69.04%	83.33%	33.33%
GNINA 1.3 rigid (re-docking) (42)	26.19%	14.28%	21.42%	9.52%

a In column 1, the values represent the total number of docking results obtained from the different tools, relative to the number present in the training data.

Collectively, these observations indicate that extensive training-set exposure alone is insufficient to achieve near-perfect pose prediction. Even under highly favourable conditions with substantial overlap between training and benchmark datasets, all DL methods continued to exhibit notable prediction failures. The most striking observation was the consistent superiority of AlphaFold 3 over Boltz-2 despite the latter having access to a larger and more recent structural corpus. This finding suggests that architecture design and confidence estimation contribute more strongly to predictive success than training-set size alone.

The difference became particularly evident when comparing minimum HA-RMSD poses with top-ranked predictions. Boltz-2 frequently sampled native-like conformations but often failed to rank them as its highest-confidence prediction. AlphaFold 3, in contrast, consistently aligned prediction confidence with structural accuracy, indicating superior confidence calibration and pose-ranking capability. DiffDock displayed similar behaviour, with acceptable minimum HA-RMSD poses occasionally generated but inconsistently ranked, resulting in larger differences between best-pose and top-ranked performance. These findings emphasize that successful ligand docking requires both accurate pose generation and reliable confidence estimation.

Analysis based on ligand COM RMSD displacement further demonstrated that many prediction failures did not arise from complete loss of binding-site recognition. For both GSK-3β and BACE1, AlphaFold 3 exhibited low COM RMSD based failure rates, indicating that predicted ligands generally occupied the correct binding pocket despite exceeding HA-RMSD thresholds. DiffDock showed a similar pattern for many complexes, where elevated HA-RMSD values frequently reflected orientation or conformational inaccuracies rather than complete binding-site displacement. Only a small subset of predictions exhibited large COM RMSD deviations indicative of incorrect pocket placement.

Only one GSK-3β complex (1J1B) exceeded the COM RMSD threshold of 5 Å for both AlphaFold 3 and Boltz-2, representing approximately 2% of evaluated cases. The elongated AMP–PNP ligand in this structure likely presents a particularly challenging prediction due to its extended geometry and multiple interaction regions. Although infrequent, such severe failures remain important because they may substantially affect downstream applications including virtual screening, fragment linking, and lead optimization.

Under redocking conditions, in which ligands are docked into their cognate crystallographic receptor conformations, both DiffDock and GNINA 1.3 can recover near-native ligand poses with substantially greater accuracy than observed under cross-docking. Redocking for all three targets in the training dataset using DiffDock yielded a 75% success rate under minimum-RMSD poses and also with the minimum-energy poses. GNINA 1.3 rigid docking also performed substantially better during redocking than cross-docking. These results demonstrate that both methods are capable of recovering near-native poses when the receptor conformation closely corresponds to the experimentally observed ligand-bound state.

### Out-of-distribution (OOD) structural generalization of DiffDock and GNINA 1.3 under cross-docking conditions

In machine learning terms, cross-docking represents an out-of-distribution (OOD) prediction problem because receptor conformations differ from those observed during training and from those used in conventional redocking evaluation. Consequently, learned protein–ligand interaction patterns may not extrapolate accurately beyond the structural manifold represented in the training distribution, leading to reduced docking accuracy. The clearest evidence for limited structural generalization was observed for GNINA 1.3. Under cross-docking conditions, GNINA 1.3 showed high proportions of complexes with HA-RMSD values > 2 Å across all three targets. For AChE, 100% of cross-docking results exceeded 2 Å according to Best_Pose_HA-RMSD. The corresponding values for Min_HA-RMSD_Pose and Min_COM_RMSD_Pose were 80% (Table 2). For BACE1, the corresponding cross-docking values for GNINA 1.3 were 91.00%, 62.82%, 88.46%, and 43.58%, while GSK-3β showed 95.23%, 69.04%, 83.33%, and 33.33%, respectively (Tables 3 and 4). These results demonstrate a pronounced deterioration in GNINA's ability to recover near-native poses when ligands are docked into non-cognate receptor conformations. DiffDock exhibited a substantially less severe but still measurable effect of cross-docking. For AChE, 20% of cross-docking results exceeded 2 Å for Best_Pose_HA-RMSD and Best_Pose_COM_RMSD, while Min_HA-RMSD_Pose and Min_COM_RMSD_Pose remained at 0% (Table 2). For BACE1, the corresponding values were 20.62%, 8.12%, 6.56%, and 1.56%, respectively (Table 3). GSK-3β showed 16.67%, 16.67%, 22.22%, and 11.11% (Table 4). Thus, although DiffDock was considerably more robust than GNINA 1.3 under cross-docking conditions. The contrast between the two methods is particularly evident when considering the Best_Pose_HA-RMSD metric. GNINA 1.3 produced >2 Å deviations in approximately 95–100% of cross-docking cases across the three targets, whereas DiffDock produced such deviations in approximately 17–21% of cases. This indicates that receptor conformational and change in ligand coordinates mismatch has a much stronger impact on GNINA 1.3 under the evaluated rigid cross-docking protocol. Nevertheless, the lower failure rates observed for DiffDock should not be interpreted as complete structural generalization, since measurable increases in HA-RMSD >2 Å were still observed for several targets and metrics. Overall, these results indicate that both GNINA 1.3 and DiffDock are affected by receptor conformational variation, but to markedly different extents. GNINA 1.3 exhibits a pronounced loss of structural robustness under cross-docking, whereas DiffDock retains substantially better performance but still shows evidence of OOD degradation. Receptor mismatch between docking receptors and experimentally observed ligand-bound conformations is therefore likely to be an important contributor to the observed errors, although limitations in conformational sampling and pose ranking may also contribute. The marked difference between conventional redocking and cross-docking performance highlights the importance of evaluating docking methods under realistic receptor-flexibility scenarios rather than relying solely on cognate–receptor redocking benchmarks. Cross-docking provides a more stringent assessment of structural robustness because it tests whether a model can transfer learned protein–ligand interaction patterns across alternative receptor conformations. The present results particularly demonstrate that apparently strong docking performance under redocking does not necessarily imply robust generalization to structurally perturbed receptor states.

Taken together, these results demonstrate three key principles. First, extensive training-test overlap does not guarantee near-perfect performance, indicating that current models remain limited in their ability to generalize across structurally diverse protein–ligand complexes. Second, architecture design, confidence calibration, and pose-ranking strategies can substantially influence predictive success beyond training-set size alone. Third, evaluation protocol strongly influences apparent performance, with cross-docking exposing substantial structural generalization failures that may remain hidden in conventional redocking benchmarks. In particular, the very high failure rates with pose RMSD >2 Å observed for GNINA 1.3 under cross-docking demonstrate the extent to which receptor conformational variation as well as change in ligand coordinates can affect docking accuracy. Future benchmarking studies should therefore prioritize (i) minimizing training-test leakage at both target and ligand levels, (ii) incorporating realistic cross-docking scenarios involving structurally diverse receptor conformations, and (iii) systematically quantifying rare but consequential failure modes, including large HA-RMSD and COM RMSD deviations. Such leakage-resistant and structure-diverse benchmarks will provide a more rigorous assessment of genuine structural generalization and facilitate the development of models that better capture the underlying determinants of protein–ligand recognition.

### Statistical analysis for cases in training data

To determine whether differences in prediction accuracy reflected systematic methodological differences rather than random variation, paired Wilcoxon signed-rank tests were applied to HA-RMSD distributions and McNemar's tests to success rates (HA-RMSD ≤ 2 Å), with Holm correction for multiple comparisons. Receptor conformations were selected *a priori* for docking analyses, using holo structures for AChE and BACE1 and an apo structure for GSK-3β.

Under the minimum HA-RMSD criterion, which assesses the best available pose for each complex rather than prospective pose-selection performance, the four methods can be compared directly under the shared crossdock condition, the only condition in which AlphaFold 3 and Boltz-2 were evaluated (Table S8). AlphaFold 3 achieved the lowest mean HA-RMSD (0.76 ± 1.25 Å; *N* = 127; 92.1% success), followed by Boltz-2 (1.07 ± 1.59 Å; *N* = 95; 89.5%), DiffDock (1.26 ± 1.39 Å; *N* = 92; 93.5%), and GNINA 1.3 (Rigid) (3.97 ± 2.56 Å; *N* = 125; 13.6%). Thus, the mean HA-RMSD ranking was AlphaFold 3 < Boltz-2 < DiffDock < GNINA 1.3, while AlphaFold 3 and DiffDock showed essentially indistinguishable success rates, and GNINA 1.3 was a clear outlier. Because GNINA 1.3 and DiffDock were also evaluated under redock (Table S8), whereas AlphaFold 3 and Boltz-2 were evaluated in a receptor-independent setting, crossdock provides the fairest common basis for comparison; pooled redock and crossdock results are therefore not reported.

GNINA 1.3 exhibited a pronounced dependence on receptor conformation, performing substantially better under redocking (1.11 ± 1.13 Å; 87.3% success) than under crossdocking (3.97 ± 2.56 Å; 13.6% success). DiffDock was comparatively robust, with HA-RMSDs of 0.84 ± 0.76 Å and 1.26 ± 1.39 Å and success rates of 93.4% and 93.5% under redock and crossdock, respectively (Table S8).

Using each method's native default pose-ranking strategy produced the same qualitative pattern, but with lower success rates than minimum HA-RMSD selection. DiffDock achieved 1.23 ± 1.55 Å and 87.67% success under redock *versus* 1.92 ± 1.92 Å and 78.87% under crossdock, whereas GNINA 1.3 deteriorated from 2.22 ± 2.57 Å and 71.4% success to 6.17 ± 3.67 Å and 8.0%, respectively. AlphaFold 3 (1.08 ± 1.71 Å; 88.2%) and Boltz-2 (1.62 ± 1.99 Å; 82.1%) were evaluated only in the receptor-independent setting. These results highlight the effect of the pose-ranking strategy on estimated performance. Because AlphaFold 3, Boltz-2, and DiffDock do not provide conventional docking energies, their first-ranked poses were used, whereas GNINA 1.3 poses were ranked by docking score. The resulting comparisons, therefore, reflect each method's native selection strategy rather than a common physical binding-energy criterion.

For the minimum HA-RMSD crossdock dataset, all six pairwise Wilcoxon comparisons among the four methods were significant after Holm correction: AlphaFold 3 differed from DiffDock (*N* = 92, *W* = 615, *P* = 8.85 × 10^−9^), GNINA 1.3 (*N* = 125, *W* = 66, *P* = 8.64 × 10^−21^), and Boltz-2 (*N* = 94, *W* = 1121.5, *P* = 5.59 × 10^−5^); DiffDock differed from GNINA 1.3 (*N* = 92, *W* = 31, *P* = 1.12 × 10^−15^) and Boltz-2 (*N* = 66, *W* = 662.5, *P* = 4.66 × 10^−3^); and GNINA 1.3 differed from Boltz-2 (*N* = 93, *W* = 246, *P* = 4.30 × 10^−13^) (Table S9). The redock comparison between DiffDock and GNINA 1.3 was not significant (*N* = 90, *W* = 2026.5, *P* = 0.93). These tests indicate differences in paired HA-RMSD distributions rather than differences in mean HA-RMSD alone.

McNemar's tests revealed a different pattern. Under minimum HA-RMSD selection, all comparisons involving GNINA 1.3 were significant: AlphaFold 3 *versus* GNINA 1.3 (*P* = 1.24 × 10^−26^), DiffDock *versus* GNINA 1.3 (*P* = 2.65 × 10^−22^), and GNINA 1.3 *versus* Boltz-2 (*P* = 2.72 × 10^−16^). No other crossdock comparison was significant: AlphaFold 3 *versus* DiffDock (*P* = 1.00), AlphaFold 3 *versus* Boltz-2 (*P* = 1.00), and DiffDock *versus* Boltz-2 (*P* = 0.094). The redock DiffDock-versus-GNINA 1.3 comparison was also non-significant (*P* = 1.00). The same pattern held under default ranking. Wilcoxon tests remained significant for all crossdock pairs except DiffDock *versus* Boltz-2 (*P* = 0.083), while McNemar tests were significant only for comparisons involving GNINA 1.3 (AlphaFold 3 *versus* GNINA 1.3, *P* = 3.23 × 10^−27^; DiffDock *versus* GNINA 1.3, *P* = 7.11 × 10^−15^; GNINA 1.3 *versus* Boltz-2, *P* = 2.11 × 10^−17^). All other McNemar comparisons had Holm-adjusted *P* ≥ 0.63.

Overall, these results demonstrate that the apparent performance differences among the four methods depend strongly on both receptor conformation and pose-selection strategy. Under the common crossdock condition, AlphaFold 3 achieved the lowest mean HA-RMSD, while DiffDock showed a comparable success rate and substantially greater robustness to receptor conformation than GNINA 1.3. The paired statistical analyses further indicate that differences in HA-RMSD distributions were generally significant, whereas success-rate differences were driven primarily by the poor crossdock performance of GNINA 1.3. The similar McNemar results for AlphaFold 3, DiffDock, and Boltz-2 also emphasize that mean HA-RMSD and success rate capture distinct aspects of predictive performance. Finally, the deterioration observed with native pose-ranking strategies highlights the importance of pose selection in evaluating docking accuracy. Taken together, these findings support crossdock as the most appropriate common basis for comparing these methods and underscore the need to consider both receptor dependence and pose-ranking strategy when interpreting structure-prediction and docking performance.

## Discussion

Recent benchmarking studies have demonstrated both the promise and limitations of AI-driven docking and co-folding approaches. While methods such as AlphaFold 3, DiffDock, SurfDock, and related frameworks often achieve strong average performance, several large-scale evaluations have shown persistent challenges related to physical plausibility, receptor conformational variability, binding-pocket novelty, and dependence on training-data similarity.^14,20–24^ However, these studies have primarily assessed performance across large heterogeneous datasets, providing limited insight into how specific binding-site architectures influence docking success or failure. Moreover, the unequal representation of protein targets in large benchmark datasets can complicate cross-target comparisons if not appropriately normalized. In the present study, docking performance for each method was therefore evaluated independently for each target using target-specific benchmark datasets, and success rates were reported as percentages relative to the total number of complexes for that target. This normalization enables fair comparison of docking methods despite differences in dataset size while allowing the influence of binding-site architecture on docking performance to be assessed systematically.

The present study addresses this gap by examining docking performance across three AD targets representing distinct cavity topologies. Rather than evaluating methods solely through aggregate success rates, our results demonstrate that receptor conformational preference and docking reliability are strongly dependent on binding-site architecture. Quantitative comparison of representative apo and holo receptor structures showed that the impact of receptor selection is highly target dependent. Conventional docking methods generally achieved higher success rates with the holo conformations of AChE and BACE1, whereas the apo conformation consistently produced superior performance for GSK-3β whereas for DiffDock cross-docking apo structures yielded better accuracy. These findings indicate that receptor state can substantially influence docking performance, but the extent of this effect depends on both the target protein and the docking algorithm employed.

One limitation of the present study is that cross-docking was performed using a single apo and a single holo receptor structure for each target. These receptor structures were selected based on the highest available X-ray resolution and minimal missing residues near the ligand-binding site to ensure consistent benchmarking. Comparison of the representative apo and holo receptors demonstrates that receptor conformation can substantially influence absolute docking success rates, with the magnitude of this effect varying across targets and docking methods. Because individual receptor structures capture only a subset of the conformational landscape, future studies employing larger ensembles of apo and holo structures will provide a more comprehensive assessment of receptor-template sensitivity and conformational robustness.

Another limitation of this study is that crystallographic water molecules and ions were removed from all receptor structures before docking to maintain a standardized receptor representation across all seven methods. This preprocessing was necessary because the conventional and AI-based methods evaluated in this study do not provide a consistent framework for incorporating explicit crystallographic waters and ions across their respective workflows. Although this approach enables a fair and uniform comparison, it reduces the biological realism of binding sites in which conserved or bridging water molecules and structural ions contribute to protein–ligand recognition. Such interactions may be relevant in systems including AChE and BACE1 and, consequently, their removal may have affected the performance of some docking methods. Explicit-solvent molecular dynamics simulations provide a complementary approach for characterizing dynamic water-mediated interactions, which can subsequently be investigated using trajectory-based structural analyses and, where appropriate, MM-GBSA/PBSA or other free-energy analysis approaches. Future benchmarking studies incorporating standardized treatment of explicit solvent and structurally relevant ions across docking methods, as such capabilities become available, would provide a more comprehensive assessment of docking performance.

The target-dependent effects observed in this study further suggest that receptor selection should be considered in the context of binding-site topology rather than treated as a universal methodological choice. Enclosed binding sites, such as the AChE gorge and the BACE1 flap pocket, showed distinct receptor-state preferences in the present benchmark, whereas the more solvent-accessible GSK-3β binding pocket exhibited a greater dependence on receptor conformation. This difference indicates that the relationship between pocket topology and receptor state is not necessarily uniform across docking methods or targets. To further elucidate the structural basis of this behavior, we compared the holo (1J1B) and apo (1I09) GSK-3β structures by examining activation-loop conformation, binding-pocket geometry, and crystallographic hydration. The analysis suggests that pocket expansion results primarily from subtle local structural rearrangements rather than large activation-loop movements, while differences in crystallographic hydration alone do not appear sufficient to explain the observed docking performance (Note S1 and Table S3).

**Fig. 7:**
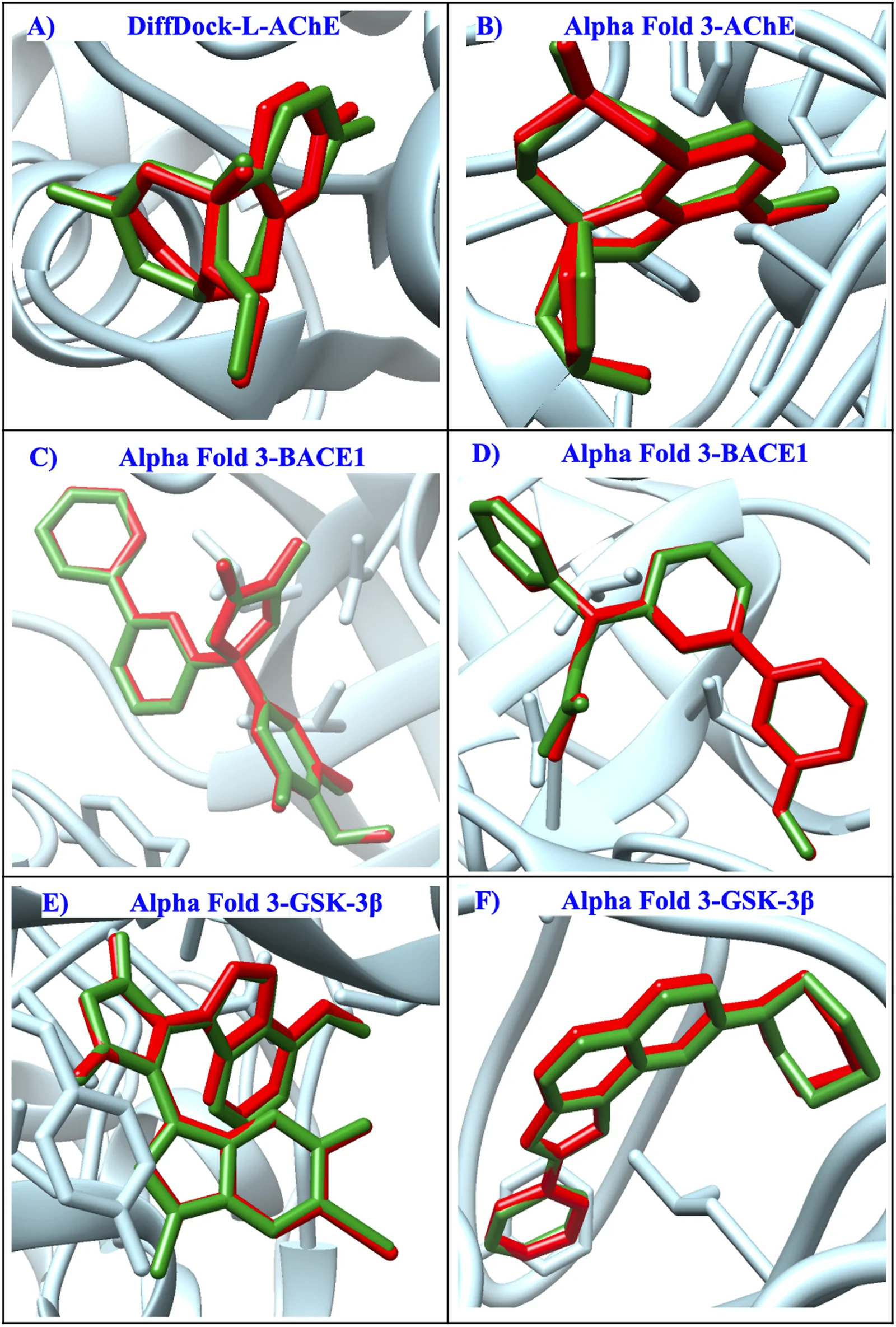
Cross-docking pose comparison across targets and methods. Superposition of docked ligand poses (red) with reference experimental conformations (green) for cross-docking across three targets: AChE, BACE1, and GSK-3β. Panels (A, C and E) represent poses with the minimum HA-RMSD, while panels (B, D and, F) show the best-scoring HA-RMSD poses selected from all evaluated docking methods.

**Fig. 8:**
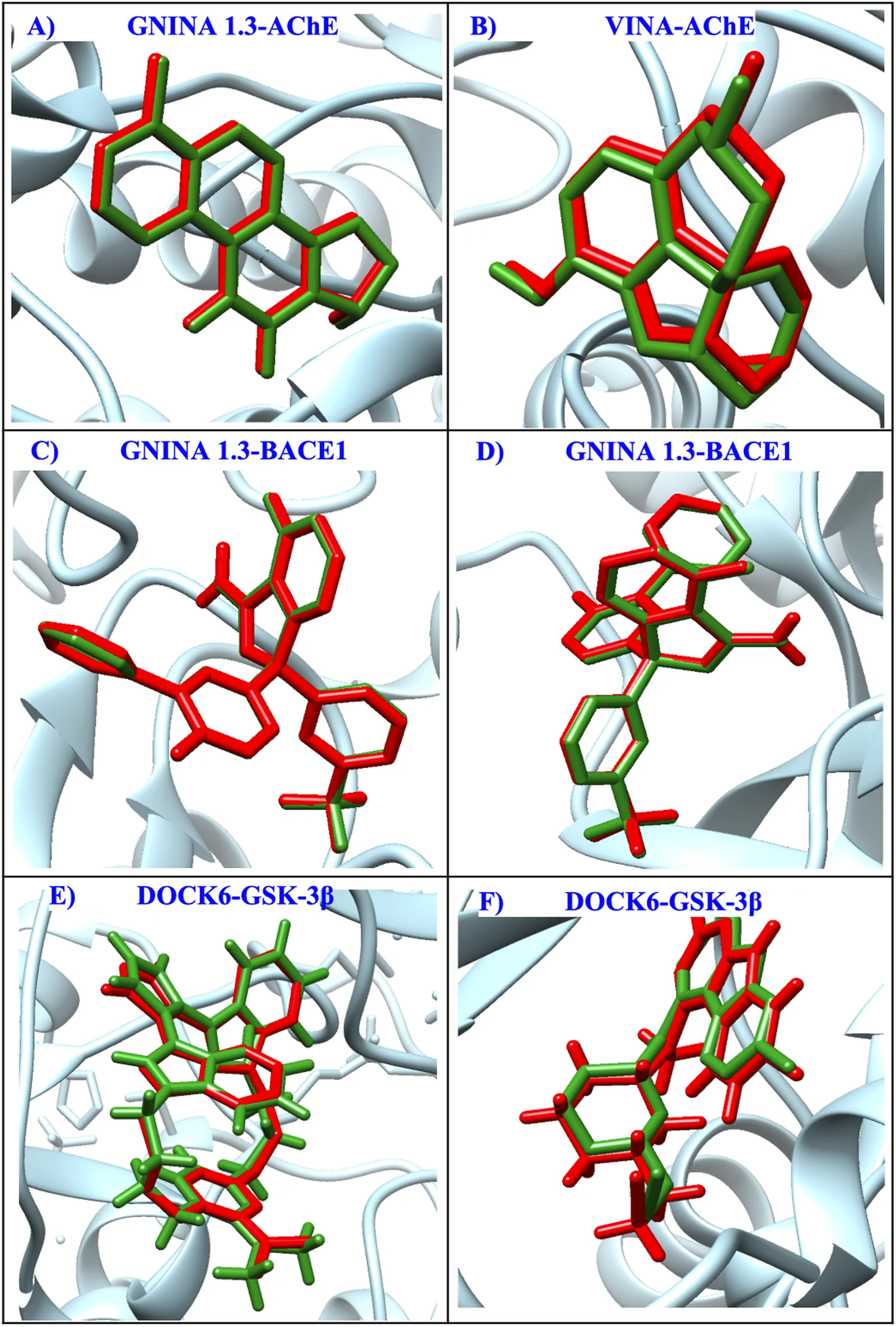
Re-docking pose comparison across targets and docking methods. Superposition of experimentally determined ligand poses (green) and corresponding re-docked ligand poses (red) for three targets: AChE, BACE1, and GSK-3β. Panels (A, C and E) show the re-docked poses with the lowest HA-RMSD relative to the corresponding experimental poses, whereas panels (B, D and F) show the highest-ranked poses based on the docking score, together with their corresponding HA-RMSD values.

Our results also highlight the limitations of relying exclusively on redocking benchmarks. Several AI-based approaches (GNINA 1.3) that performed well during redocking exhibited substantial declines under cross-docking conditions, indicating that successful prediction on cognate receptor-ligand complexes does not necessarily translate into robust generalisation across receptor conformations. This finding is consistent with recent reports that training-data similarity alone is insufficient to ensure reliable docking performance on structurally distinct binding environments^14,21–24^ (Fig. 7 and 8). Cross-docking therefore provides an important complementary test of whether docking methods can generalize beyond the receptor–ligand structural context represented by the cognate complex.

An additional observation is that flexible receptor docking did not consistently improve docking accuracy. In the present study, receptor flexibility was limited to a selected subset of binding-site side chains to maintain computational feasibility across the benchmark dataset. Consequently, flexible docking sampled side-chain conformations while the protein backbone remained fixed in the starting configuration. This restricted conformational sampling likely contributed to the narrowly distributed pocket volumes observed for flexible docking. These findings suggest that the quality and extent of conformational sampling may be more important than the inclusion of limited receptor flexibility alone, particularly for targets that undergo substantial ligand-induced conformational changes.

Taken together, these findings indicate that docking success is determined not only by the underlying algorithm but also by the structural characteristics of the target binding site. By directly linking docking performance to cavity topology and receptor conformational state, this study provides a mechanistic perspective that complements recent large-scale benchmarking efforts and offers practical guidance for selecting docking strategies in structure-based drug discovery. More broadly, our findings emphasize that docking performance should be interpreted as an interaction between the algorithm, receptor conformational state, and binding-site architecture rather than as an intrinsic property of the docking method alone.

A promising direction for future research is the development of hybrid AI-physics workflows that combine the complementary capabilities of modern AI-based structure prediction with physics-based computational methods. In the present benchmark, AI-based co-folding approaches outperformed the physics-based docking methods for binding-pose prediction, despite the latter incorporating physics-based scoring functions that account for energetic and solvation effects. Therefore, combining physics-based scoring with AI-generated poses may not necessarily provide further improvements in pose prediction over current AI-based approaches. However, such hybrid strategies may be particularly valuable for binding-affinity prediction, where currently available AI-based co-folding methods generally do not directly estimate the free energy of binding. In this context, AI-based methods could be used to generate plausible protein–ligand complexes, followed by physics-based rescoring or more rigorous free-energy calculations to estimate binding energetics. Although such hybrid workflows were beyond the scope of the present benchmark, their evaluation could provide a useful direction for integrating AI-based structure prediction with physics-based approaches, particularly for improving the estimation of binding affinity in structure-based drug discovery.

## Conclusion

This study demonstrates that docking performance across Alzheimer's disease targets is governed by the interplay of receptor geometry, binding-site architecture, model design, and scoring strategy rather than by training-data exposure alone. The results highlight the advantages of modern AI-driven approaches for handling receptor and ligand variability while revealing persistent challenges in structural generalization and pose ranking. Receptor conformation emerged as a critical determinant of success, with docking outcomes strongly influenced by target-specific cavity characteristics, emphasizing the importance of receptor selection prior to virtual screening. Under the limited side-chain flexibility protocol evaluated here (due to high computation cost), the superiority of rigid over flexible docking further indicates that accurate treatment of receptor flexibility remains a major unresolved challenge. Collectively, this work establishes a comprehensive benchmarking framework that links docking performance to structural variability and provides a foundation for evaluating next-generation docking methods across diverse therapeutic targets.

## Author contributions

Kapali Suri: data curation, formal analysis, investigation, methodology, software & visualisation, validation, writing—original draft, writing—review and editing. Anshul Yadav: formal analysis, software & visualization. Abhishek Tripathi: formal analysis. N. Arul Murugan: Conceptualisation, investigation, project administration, supervision, funding acquisition writing—review and editing.

## Conflicts of interest

The authors declare no conflict of interest. The research was conducted in the absence of any commercial or financial relationships that could be construed as a potential conflict of interest.

## Supplementary Material

RA-OLF-D6RA05440D-s001

## Data Availability

All protein structures used in this study correspond to acetylcholinesterase (AChE), β-secretase 1 (BACE1), and glycogen synthase kinase-3β (GSK-3β) with Uniprot ID: P22303, UniProt ID: P56817 and Uniprot ID: P49841. The list of PDB IDs was programmatically retrieved from the EBI Proteins API and cross-checked with the RCSB Protein Data Bank (https://www.rcsb.org). Docking calculations were performed with freely available Software: AutoDock (http://autodock.scripps.edu), AutoDock Vina (http://vina.scripps.edu), DOCK 6 (http://dock.compbio.ucsf.edu), GNINA 1.3 (https://github.com/gnina/gnina), DiffDock (https://github.com/gcorso/DiffDock), Boltz-2 (https://github.com/jwohlwend/boltz), and AlphaFold 3 (https://github.com/google-deepmind/alphafold3). All molecular docking input files, receptor and ligand structures, pose prediction outputs, and custom scripts used for pose analysis are available in https://www.doi.org/10.6084/m9.figshare.33321408. Supplementary information (SI): detailed statistical analyses, docking performance results, methodological/reproducibility information, structure comparisons and supporting figures for the main study. See DOI: https://doi.org/10.1039/d6ra05440d.
