# Supplementary material for: Cross-docking and redocking reveal distinct determinants of success in physics-based and AI-driven binding pose prediction in protein–ligand complexes
Source: RSC Adv. 2026 Sep 8;16(43):53425–44. doi: 10.1039/d6ra05440d (PMC13551558; doi:10.1039/d6ra05440d)
Supplement: Supplementary file 1 [file RA-016-D6RA05440D-s001.pdf]

Supplementary Information (SI) for RSC Advances.
This journal is © The Royal Society of Chemistry 2026

Supplementary File

Table S1. Clarification of Software Versions, Input Parameters, Ranking Criteria, and Reproducibility for AlphaFold 3, Boltz-2, GNINA 1.3, and DiffDock-L

Method	Version	Protein Input	Ligand Input	Settings Used	Reproducibility
AlphaFold 3	3	Protein amino acid sequence	SMILES	Default inference settings	https://github.com/google-deepmind/AlphaFold3
Boltz-2	2	Protein amino acid sequence	SMILES	Default inference settings	https://github.com/jwohlwend/Boltz-2
DiffDock	February 2024	Protein PDB	SMILES	Default inference settings	https://github.com/gcorso/DiffDock
GNINA 1.3	1.3	Protein PDB	Ligand SDF	Default settings with CNN refinement enabled	https://github.com/gnina/gnina

Table S2. Heavy-atom RMSD and heavy-atom center-of-mass (COM) RMSD < 2 Å for all three targets.

ACETYLCHOLINESTERASE												
**Tool (Docking Mode)**	RMSD Min%	RMSD Best%	COM Min%	COM Best%								
**Boltz-2**	77	45	90	72								
**AlphaFold 3**	52	52	84	68								
	Holo RMSD Min%	Holo RMSD Best%	Holo COM Min%	Holo COM Best%	Apo RMSD Min%	Apo RMSD Best%	Apo COM Min%	Apo COM Best%	Redocking RMSD Min%	Redocking RMSD Best%	Redocking COM Min%	Redocking COM Best%
**DiffDock**	52	42	78	63	63	36	73	52	62	62	75	68
**DOCK6 (Rigid)**	0	0	0	0	6	0	43	37	55	44	72	55

**AutoDock Vina (Rigid)**	21	5	68	47	0	0	47	26	71	57	85	71
**AutoDock Vina (Flexible)**	22	9	63	27	0	0	31	9	27	18	72	40
**AutoDock4 (Rigid)**	15	0	40	20	0	0	35	15	61	23	85	57
**AutoDock4 (Flexible)**	0	0	15	5	0	0	0	0	20	5	90	30
**GNINA 1.3 (Rigid)**	10	5	60	15	5	0	15	10	76	52	100	80
**GNINA 1.3 (Flexible)**	28	21	85	50	0	0	50	14	10	10	75	35
**BETA-SECRETASE_1**												
**Tool (Docking Mode)**	RMSD Min%	RMSD Best%	COM Min%	COM Best%								
**AlphaFold 3**	94	91										
**Boltz-2**	90	83										
	Holo RMSD Min%	Holo RMSD Best%	Holo COM Min%	Holo COM Best%	Apo RMSD Min%	Apo RMSD Best%	Apo COM Min%	Apo COM Best%	Redocking RMSD Min%	Redocking RMSD Best%	Redocking COM Min%	Redocking COM Best%
**DiffDock**	88	75	96	91	86	73	97	88	91	88	98	95
**DOCK6 (Rigid)**	0	0	0	0	0	0	17	8	62	54	81	72
**AutoDock Vina (Rigid)**	9	2	73	31	18	9	53	33	56	41	81	62
**AutoDock Vina (Flexible)**	10	3	76	36	1	0	8	2	11	2	69	26
**AutoDock4 (Rigid)**	22	12	80	46	7	3	59	26	48	35	78	53
**AutoDock4 (Flexible)**	0	0	10	6	0	0	1	0	5	0	83	26
**GNINA 1.3 (Rigid)**	14	4	74	42	13	6	52	33	89	76	95	86
**GNINA 1.3 (Flexible)**	4	2	39	6	2	1	28	4	7	1	39	4
**GLYCOGEN_SYNTHASE_KINASE-3_BETA**												
**Tool (Docking Mode)**	RMSD Min%	RMSD Best%	COM Min%	COM Best%								
**AlphaFold 3**	94	91	97	94								
**Boltz-2**	87	86	92	90								

Tool (Docking Mode)	Holo RMSD Min%	Holo RMSD Best%	Holo COM Min%	Holo COM Best%	Apo RMSD Min%	Apo RMSD Best%	Apo COM Min%	Apo COM Best%	Redocking RMSD Min%	Redocking RMSD Best%	Redocking COM Min%	Redocking COM Best%
**DiffDock**	66	55	88	77	77	66	88	88	83	83	100	83
**DOCK6 (Rigid)**	7	4	38	15	10	4	55	23	85	73	95	83
**GNINA 1.3 (Rigid)**	0	0	0	0	23	10	73	50	80	58	97	88
**AutoDock4 (Rigid)**	0	0	0	0	17	10	82	35	67	35	89	70
**AutoDock Vina (Rigid)**	0	0	0	0	11	1	82	29	62	31	95	79
**AutoDock Vina (Flexible)**	0	0	0	0	0	0	47	10	7	1	49	29
**AutoDock4 (Flexible)**	0	0	0	0	0	0	2	2	2	1	88	26
**GNINA 1.3 (Flexible)**	0	0	0	0	0	0	52	13	0	0	0	0

Note 1. Sequence Alignment of BACE1 Apo (PDB: 7MYI) and Holo (PDB: 3TPJ) Structures Showing Residue Numbering Correspondence for Flexible Docking

A sequence alignment between the two structures further confirmed that the catalytic dyad and flap residues occupy equivalent structural positions, with differences restricted to the N-terminal expression tag and a small number of conservative substitutions outside the active site. During flexible receptor preparation, the flexible residues were specified using the residue identifiers present in the prepared receptor files. Since AutoDock/GNINA 1.3 flexible docking generates a separate flexible receptor by extracting the specified residues from the receptor structure, an incorrect residue number or residue identity (e.g., specifying ASP32 when the structure contains VAL32 at that position and ASP33 is the catalytic residue) would prevent the correct generation of the flexible receptor and result in an incorrect receptor definition. Therefore, residue numbering was verified prior to docking to ensure that the catalytic residues (Asp32 and Asp228) and the flap residue (Tyr71) corresponded to the intended structural positions in both receptors. We have clarified this procedure in the revised manuscript and included the sequence alignment as supporting evidence.

```
3TPJ            -RGSFVEMVDNLRGKSGQGYYVEMTVGSPPQTLNILVDTGSSNFAVGAAPHPFLHRYYQR
7MYI            RRGSFVEMVDNLRGKSGQGYYVEMTVGSPPQTLNILVDTGSSNFAVGAAPHPFLHRYYQR
                 ***********************************************************

3TPJ            QLSSTYRDLRKGVYVPYTQGAWAGELGTDLVSIPHGPNVTVRANIAAITESDKFFINGSN
7MYI            QLSSTYRDLRKGVYVPYTQGKWEGELGTDLVSIPHGPNVTVRANIAAITESDKFFINGSN
                ******************** * *************************************

3TPJ            WEGILGLAYAEIARPDDSLEPFFDSLVKQTHVPNLFSLQLCGAGFPLNQSEVLASVGGSM
7MYI            WEGILGLAYAEIARPDDSLEPFFDSLVKQTHVPNLFSLQLCGAGFPLNQSEVLASVGGSM
                ************************************************************

3TPJ            IIGGIDHSLYTGSLWYTPIRREWYYEVIIVRVEINGQDLKMDCKEYNYDKSIVDSGTTNL
7MYI            IIGGIDHSLYTGSLWYTPIRREWYYEVIIVRVEINGQDLKMDCKEYNYDKSIVDSGTTNL
                ************************************************************

3TPJ            RLPKKVFEAAVKSIKAASSTEKFPDGFWLGEQLVCWQAGTTPWNIFPVISLYLMGEVTNQ
7MYI            RLPKKVFEAAVKSIKAASSTEKFPDGFWLGEQLVCWQAGTTPWNIFPVISLYLMGEVTNQ
                ************************************************************

3TPJ            SFRITILPQQYLRPVEDVATSQDDCYKFAISQSSTGTVMGAVIMEGFYVVFDRARKRIGF
7MYI            SFRITILPQQYLRPVEDVATSQDDCYKFAISQSSTGTVMGAVIMEGFYVVFDRARKRIGF
                ************************************************************

3TPJ            AVSACHVHDEFRTAAVEGPFVTLDMEDCGYNI
7MYI            AVSACHVHDEFRTAAVEGPFVTLDMEDCGYN-
                *******************************
```

Figure S1: Alignment of 7MYI & 3TPJ

Note S2. Structural comparison of the GSK-3β holo (1J1B) and apo (1I09) conformations: activation-loop dynamics, pocket geometry, and hydration analysis

The backbone RMSD of the activation loop (residues 200–226) between the holo structure (PDB: 1J1B) and the apo structure (PDB: 1I09) is only 0.26 Å, indicating that the activation loop does not undergo a substantial rigid-body displacement between the two conformations. Nevertheless, the binding-pocket volume increases from 1482.0 $Å^3$ in the holo structure to 1886.6 $Å^3$ in the apo structure. This observation suggests that the enlarged cavity is generated primarily through subtle local structural adjustments, including side-chain rotamer changes, minor backbone shifts, and small rearrangements of neighboring secondary-structure elements, rather than by large-scale movement of the activation loop itself.

To investigate whether crystallographic hydration contributes to the observed docking performance, structural water molecules surrounding the LigPlot-defined binding site were quantified for both conformations **(Table 3)**. The holo structure (PDB: 1J1B) contains 234 crystallographic water molecules, including 28 unique water molecules located within 5 Å of the binding-site residues, whereas the apo structure (PDB: 1I09) contains only 23 crystallographic water molecules, with four waters occupying the corresponding pocket region. Furthermore, twelve structural water molecules are located within 5 Å of the bound ANP ligand in the holo structure, forming an extensive hydration network involving residues such as Asp200, Lys85, Thr138, Tyr134, Asp133, Val110, Phe67, Asn64, and Gly65. In contrast, the apo conformation exhibits a comparatively sparse hydration pattern, with only a few pocket-associated waters primarily contacting Asp200, Asn186, and Phe67.

AutoDock4 and AutoDock Vina employ empirical scoring functions that approximate solvation using implicit continuum-solvent models and therefore do not explicitly represent conserved crystallographic water molecules or rigorously account for the free-energy cost associated with displacing ordered waters during ligand binding. Consequently, these methods cannot fully capture water-mediated hydrogen-bonding networks, entropic contributions arising from structural-water displacement, or the thermodynamic stabilization provided by conserved hydration sites. Such limitations may influence pose ranking and scoring, particularly in highly hydrated binding pockets.

However, the present results indicate that hydration alone cannot account for the observed docking behavior. If structural-water displacement were the dominant determinant of docking accuracy, poorer performance would be expected for the highly hydrated holo pocket (1J1B), where numerous ordered waters would require displacement upon ligand binding. Instead, the opposite trend was observed, with the more open apo conformation (1I09), despite containing substantially fewer crystallographic waters, consistently producing higher docking accuracy. These findings indicate that the observed differences in docking performance are unlikely to arise solely from desolvation effects. Rather, docking accuracy appears to reflect the combined influence of subtle local conformational differences that alter pocket geometry,

ligand accessibility, and the inherent limitations of empirical implicit-solvent scoring functions. Thus, while increased pocket accessibility expands the conformational search space available to docked ligands, geometric accessibility alone is insufficient to explain pose prediction accuracy without considering hydration effects and the approximations inherent in current docking scoring functions.

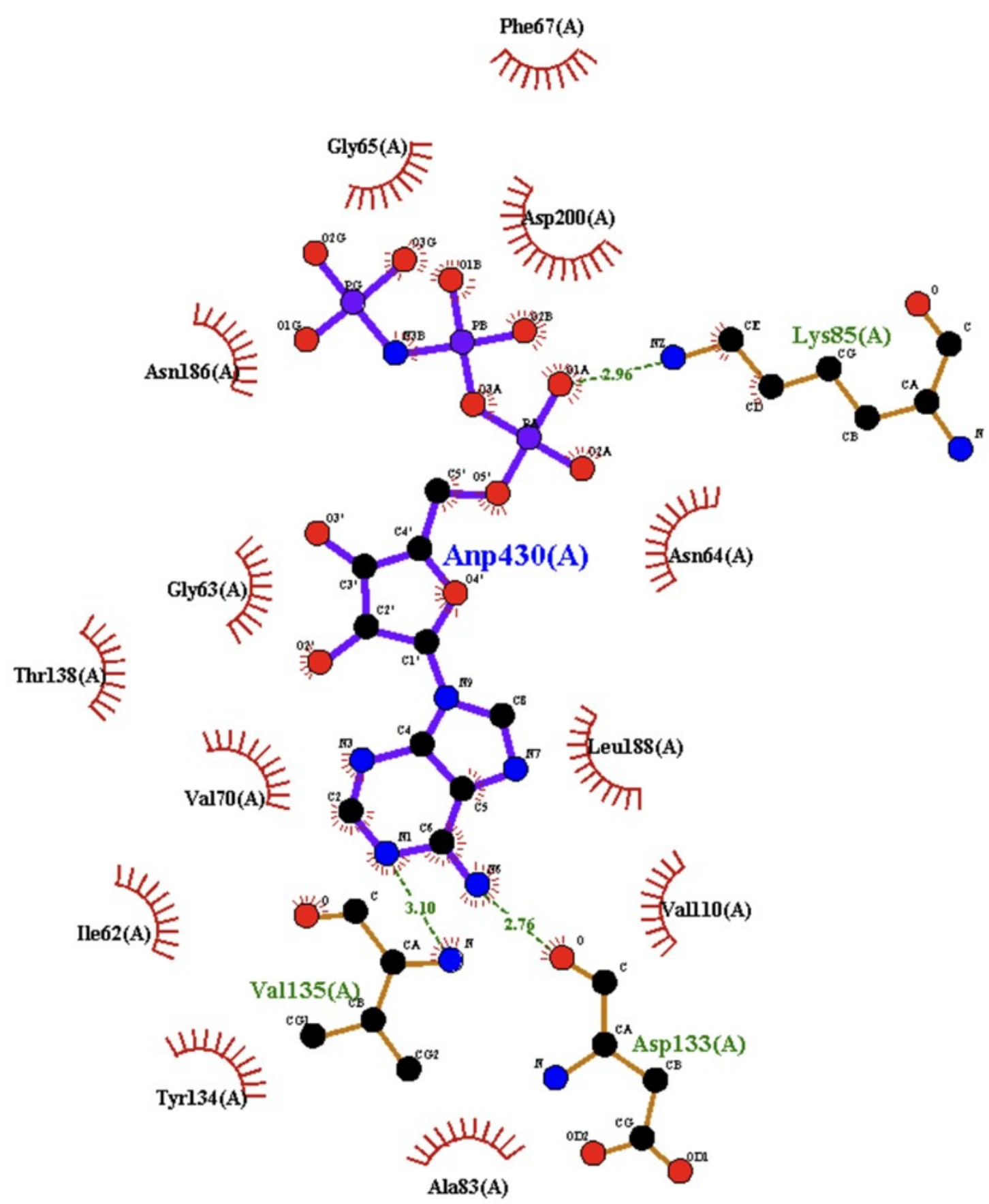

Figure S2. LigPlot Analysis of the Holo GSK-3β Structure (PDB: 1J1B) Showing Protein–Ligand Interactions Within the Binding Pocket

Table S3. Structural comparison of the GSK-3β holo (1J1B) and apo (1I09) conformations

Parameter	1J1B (Holo, ANP-bound)	1I09 (Apo, Open)
Conformational state	Holo	Apo
Pocket volume (Å³)	1482.00	1886.63
Total crystallographic water molecules	234	23
Unique pocket waters (within 5 Å of LigPlot-defined binding-site residues)	28	4
Waters within 5 Å of bound ligand	12 (ANP)	Not applicable
Pocket hydration	Extensive ordered hydration network	Sparse hydration
Representative hydrated residues	Asp200, Lys85, Thr138, Tyr134, Asp133, Val110, Phe67, Asn64, Gly65	Asp200, Asn186, Phe67
Binding-site accessibility	More compact	More open
Docking performance	Lower	Higher
Structural interpretation	Greater contribution of conserved water-mediated interactions that are not explicitly represented by AutoDock4 and AutoDock Vina implicit-solvent scoring functions	Reduced contribution of structural waters together with increased pocket accessibility is consistent with the improved docking performance observed for the apo conformation

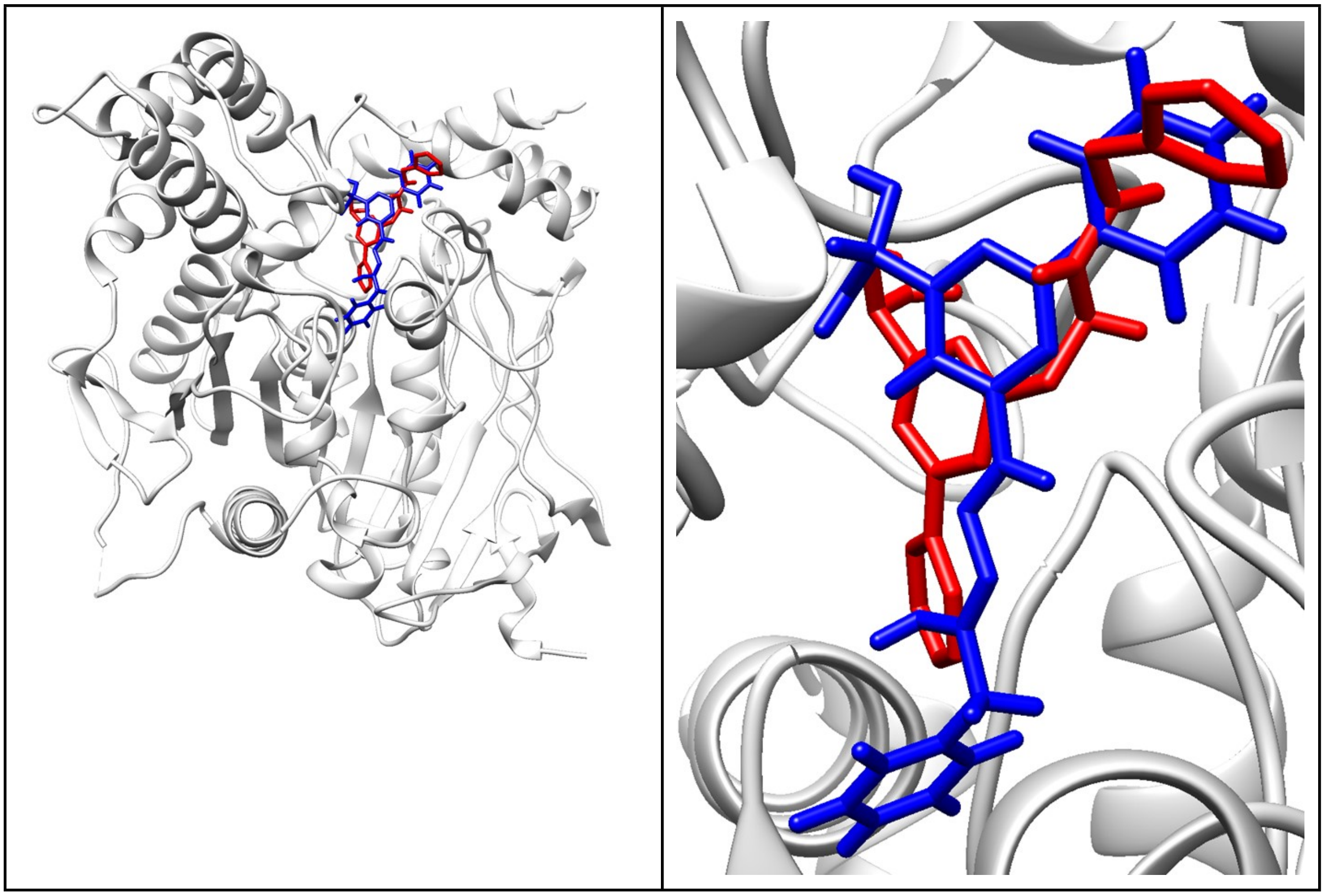

Figure S3: Representative Docking Poses with Correct Binding-Site Occupancy but Incorrect Ligand Orientation

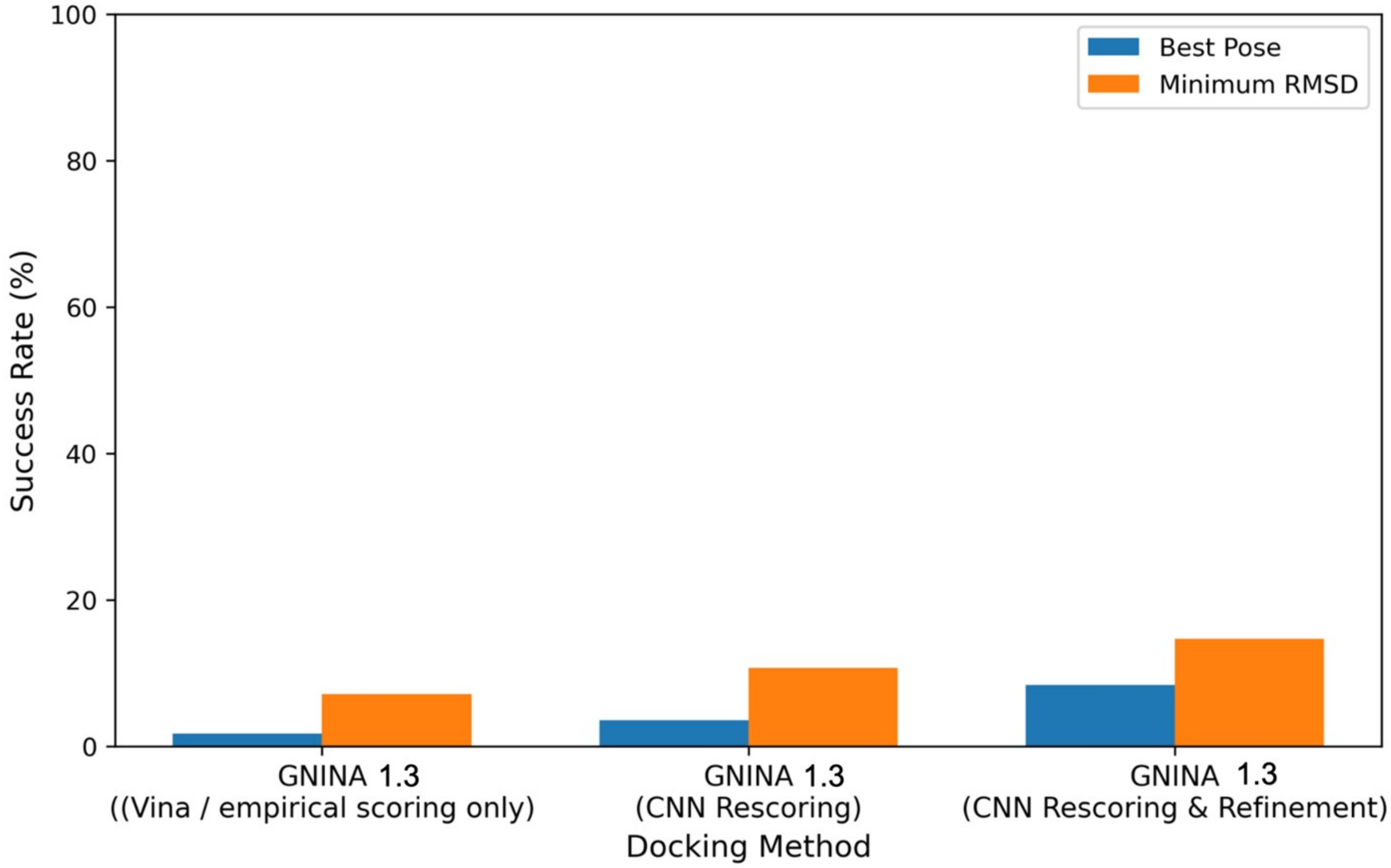

Figure S4: Comparison of Best Pose and Minimum RMSD success rates for the three GNINA 1.3 scoring configurations (empirical Vina scoring, CNN rescoring, and CNN rescoring with refinement) using the 7MYI holo receptor with cross-docked BACE1 ligands.

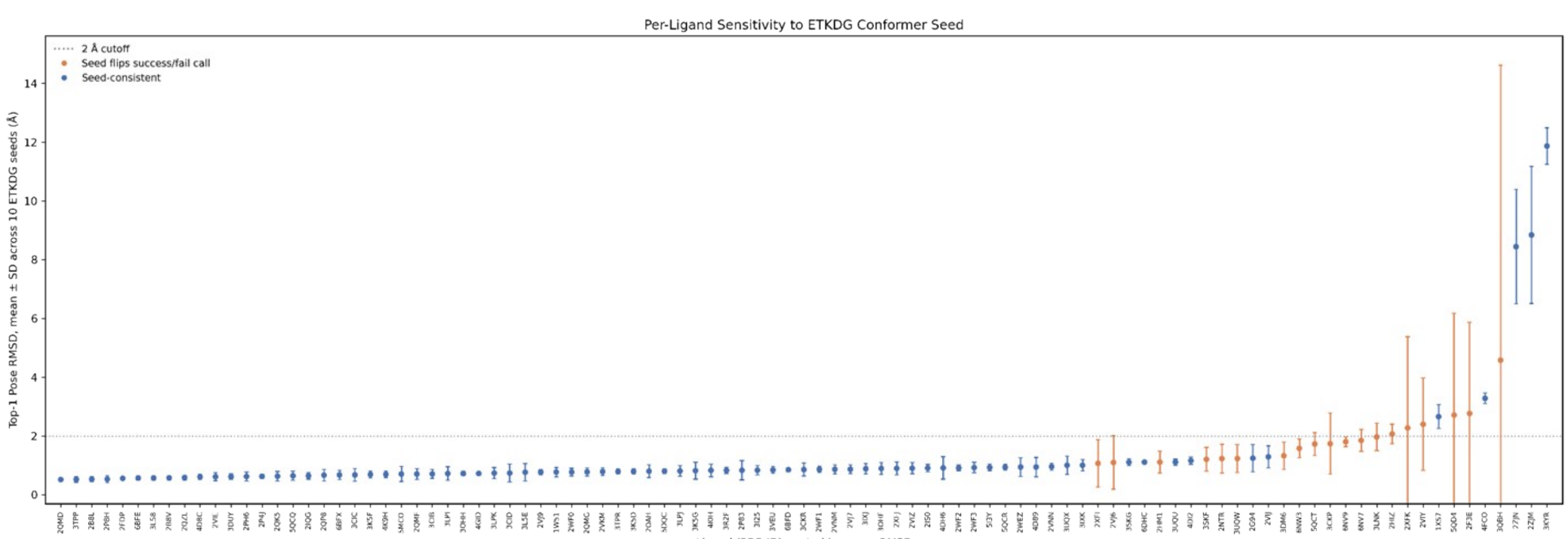

Figure S5. Histogram of within-ligand SD. Distribution of within-ligand standard deviations of top-1 RMSD across the ten ETKDG conformers, showing that most ligands exhibit low variability with a small number of highly variable outliers.

Explanation: This is the most granular view of conformer-seed variability. When ordered from lowest to highest median RMSD, the first ~85 ligands generally exhibit narrow, low-RMSD distributions, indicating that their docking outcomes are relatively consistent across conformer seeds. Toward the higher-RMSD end of the distribution, however, several ligands show substantially wider spreads, with some spanning approximately 4–11 Å across seeds. These ligands therefore contribute disproportionately to the overall seed-sensitivity signal. The variability is not uniformly distributed across the dataset but is concentrated in a relatively small subset of more challenging ligands.

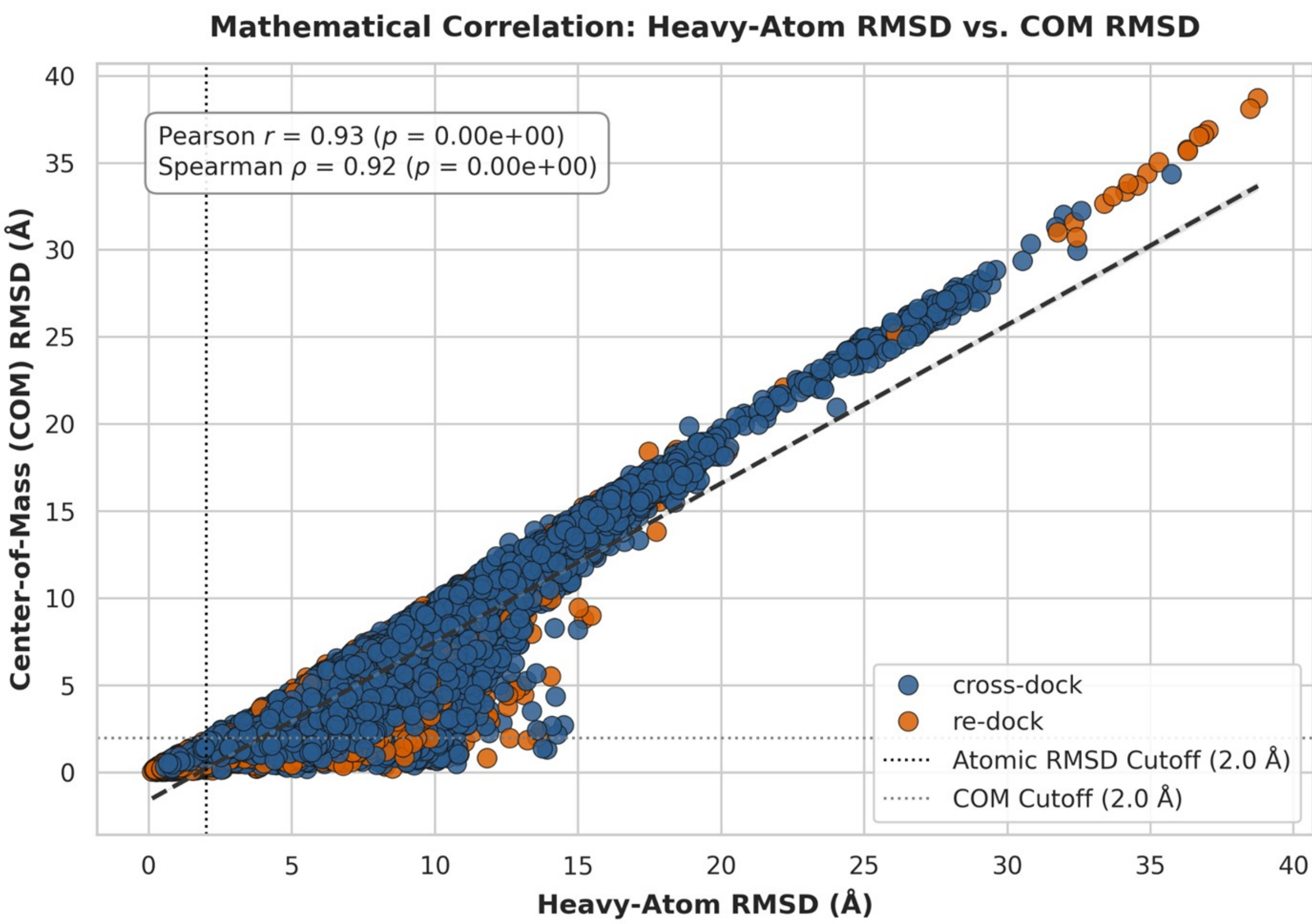

Figure S6. Correlation between heavy-atom RMSD and COM RMSD. Mathematical correlation between heavy-atom RMSD and center-of-mass (COM) RMSD across all docking poses. The strong correlation demonstrates that COM displacement increases consistently with atomic RMSD.

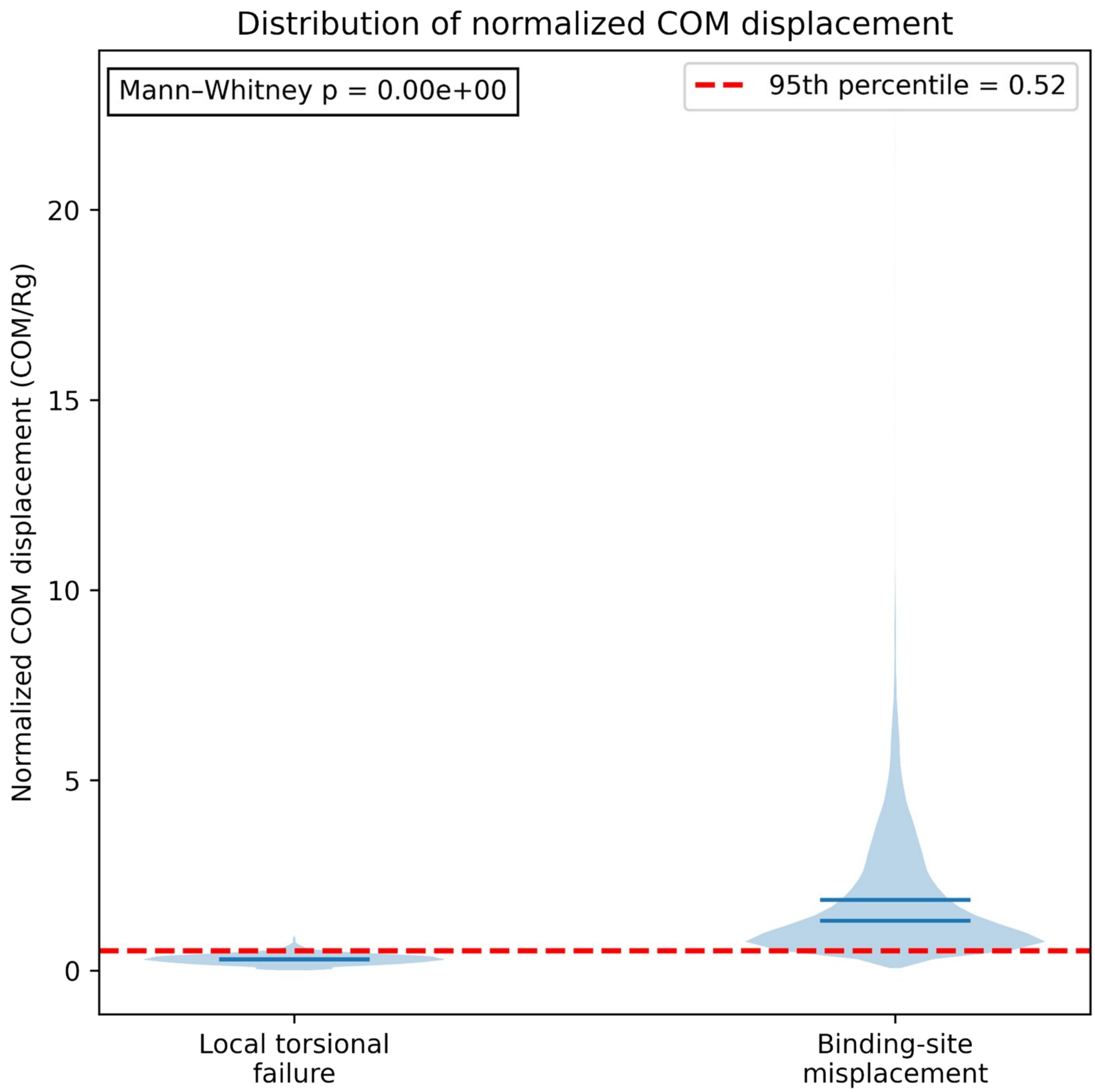

Figure S7. Distribution of radius-of-gyration-normalized COM displacement (COM/Rg) for local torsional failures (heavy-atom RMSD >2 Å and COM <2 Å) and global binding-site misplacements (heavy-atom RMSD >2 Å and COM ≥2 Å). The dashed line denotes the proposed normalized threshold (COM/Rg = 0.52), corresponding to the 95th percentile of the torsional-failure distribution.

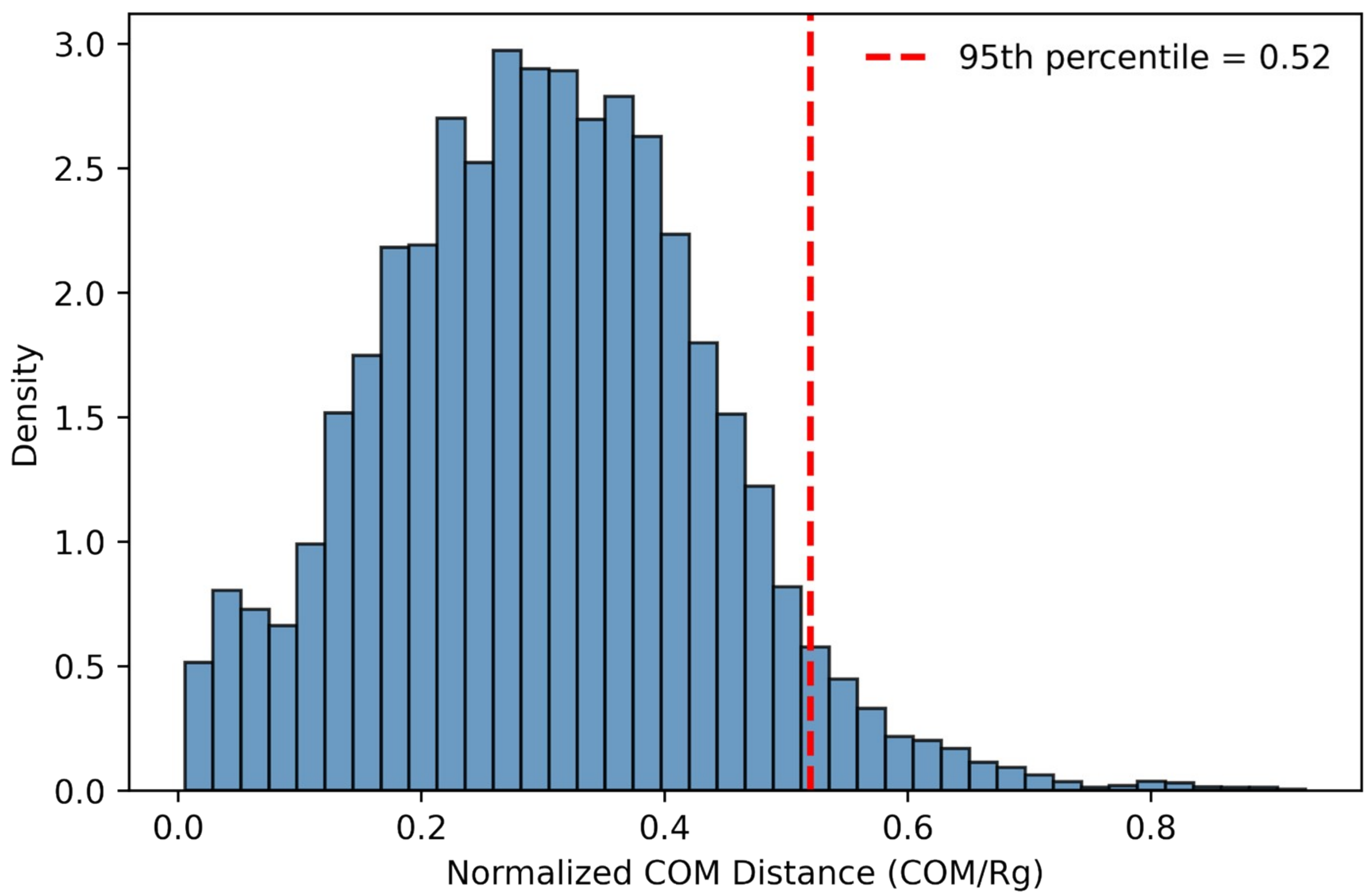

Figure S8. Distribution of normalized COM displacement (COM/Rg) for poses exhibiting local torsional deviations. The proposed normalized threshold (COM/Rg = 0.52) corresponds to the 95th percentile of this distribution.

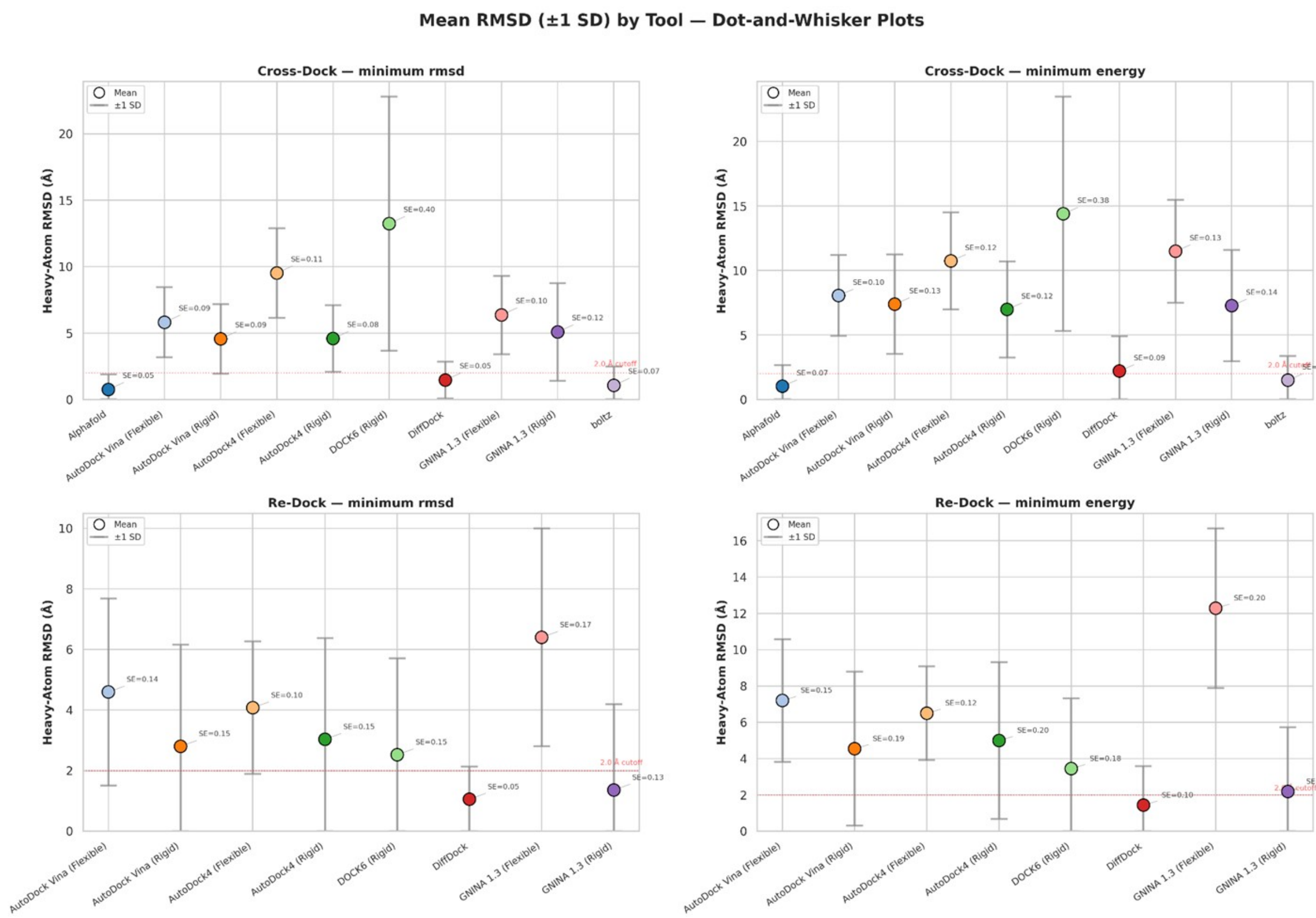

Figure S9: Mean Heavy-Atom RMSD (±1 SD) Across Docking Methods for Cross-Docking and Re-Docking

Mean heavy-atom RMSDs varied substantially across docking tools, ranging from <2 Å for DiffDock, Boltz-2, and AlphaFold 3 to >10 Å for DOCK6 (Rigid) and GNINA 1.3 (Flexible) in the cross-docking panels. The latter methods also showed the largest standard deviations (up to ~9–10 Å), indicating substantial variability among individual predictions, whereas DiffDock and Boltz-2 exhibited comparatively narrow distributions (SD <2 Å). Standard errors of the mean were small across panels (0.05–0.40 Å), suggesting reasonably precise estimates of the mean given the sample sizes. Compared with minimum-RMSD selection, minimum-energy selection generally resulted in higher mean RMSDs and greater variability for most tools, although the magnitude of this effect varied across methods.

Table S4. Crossdocking performance summary statistics across docking/scoring tools, including AlphaFold 3 and Boltz-2: sample sizes, failed-run counts, RMSD distributions, and success rates (RMSD ≤ 2.0 Å) with 95% Wilson confidence intervals.

Aggregation Type	Tool	Expected Total (N)	Valid Evaluated (n)	Failed Runs	Failure Rate (%)	Mean ± SD (Å)	Standard Error (Å)	Median (IQR) (Å)	Success Rate (≤ 2.0 Å)	95% Wilson CI
**Minimum RMSD**	AlphaFold 3	494	489	5	1.01%	1.02 ± 1.64	0.07	0.54 (0.54)	89.98%	[87.0%, 92.3%]
**Minimum RMSD**	AutoDock Vina (Flexible)	988	944	44	4.45%	8.06 ± 3.13	0.10	8.02 (3.18)	2.01%	[1.3%, 3.1%]
**Minimum RMSD**	AutoDock Vina (Rigid)	988	938	50	5.06%	7.39 ± 3.85	0.13	7.22 (4.12)	5.44%	[4.2%, 7.1%]
**Minimum RMSD**	AutoDock4 (Flexible)	988	957	31	3.14%	10.74 ± 3.77	0.12	10.57 (3.72)	0.00%	[0.0%, 0.4%]
**Minimum RMSD**	AutoDock4 (Rigid)	988	975	13	1.32%	6.98 ± 3.73	0.12	6.77 (4.46)	7.59%	[6.1%, 9.4%]
**Minimum RMSD**	DOCK6 (Rigid)	988	558	430	43.52%	14.40 ± 9.08	0.38	10.13 (17.91)	1.25%	[0.6%, 2.6%]
**Minimum RMSD**	DiffDock	988	824	164	16.60%	2.20 ± 2.70	0.09	1.34 (1.22)	72.94%	[69.8%, 75.9%]
**Minimum RMSD**	GNINA 1.3 (Flexible)	988	930	58	5.87%	11.49 ± 3.98	0.13	11.90 (5.41)	1.61%	[1.0%, 2.6%]
**Minimum RMSD**	GNINA 1.3 (Rigid)	988	940	48	4.86%	7.27 ± 4.32	0.14	6.63 (5.38)	5.53%	[4.2%, 7.2%]
**Minimum RMSD**	Boltz-2	494	424	70	14.17%	1.50 ± 1.88	0.09	0.84 (0.90)	82.31%	[78.4%, 85.6%]
**Minimum RMSD**	AlphaFold 3	494	489	5	1.01%	0.75 ± 1.14	0.05	0.43 (0.39)	93.05%	[90.4%, 95.0%]

**Minimum RMSD**	AutoDock Vina (Flexible)	988	944	44	4.45%	5.81 ± 2.63	0.09	5.54 (4.14)	5.72%	[4.4%, 7.4%]
**Minimum RMSD**	AutoDock Vina (Rigid)	988	938	50	5.06%	4.56 ± 2.62	0.09	4.21 (2.60)	12.58%	[10.6%, 14.9%]
**Minimum RMSD**	AutoDock4 (Flexible)	988	957	31	3.14%	9.51 ± 3.36	0.11	9.58 (3.36)	0.31%	[0.1%, 0.9%]
**Minimum RMSD**	AutoDock4 (Rigid)	988	975	13	1.32%	4.58 ± 2.51	0.08	4.30 (2.92)	13.64%	[11.6%, 15.9%]
**Minimum RMSD**	DOCK6 (Rigid)	988	558	430	43.52%	13.23 ± 9.56	0.40	8.42 (18.64)	2.69%	[1.6%, 4.4%]
**Minimum RMSD**	DiffDock	988	824	164	16.60%	1.46 ± 1.38	0.05	1.04 (0.81)	85.68%	[83.1%, 87.9%]
**Minimum RMSD**	GNINA 1.3 (Flexible)	988	930	58	5.87%	6.36 ± 2.95	0.10	5.83 (3.50)	3.23%	[2.3%, 4.6%]
**Minimum RMSD**	GNINA 1.3 (Rigid)	988	940	48	4.86%	5.08 ± 3.67	0.12	4.08 (3.62)	13.51%	[11.5%, 15.8%]
**Minimum RMSD**	Boltz-2	494	424	70	14.17%	1.06 ± 1.41	0.07	0.60 (0.53)	89.62%	[86.4%, 92.2%]

Table S5. Pairwise statistical comparisons of crossdocking performance between tools (Wilcoxon signed-rank and McNemar's tests, Holm-adjusted p-values)

**Aggregation Type**	**Comparison**	**Matched Pairs (n)**	**Wilcoxon W**	**Wilcoxon p-value**	**McNemar p-value**	**Wilcoxon p-value (Holm-adjusted)**	**McNemar p-value (Holm-adjusted)**
**Minimum RMSD**	AlphaFold 3 vs Boltz-2	407	18070.0	8.96041330388449E-23	0.0004908786448402840	9.85645463427294E-22	0.004908786448402840
**Minimum RMSD**	AlphaFold 3 vs AutoDock Vina (Flexible)	469	892.0	4.23469218931472E-76	3.29939413777366E-89	1.22806073490127E-74	1.18778188959852E-87
**Minimum RMSD**	AlphaFold 3 vs AutoDock Vina	469	1305.0	5.66726194451872E-75	1.96043953226206E-89	1.53016072502005E-73	7.25362626936964E-88

	(Rigid)						
**Minimum RMSD**	AlphaFold 3 vs AutoDock4 (Flexible)	474	154.0	5.9066888114363E-79	1.20500409220826E-94	2.0673410840027E-77	4.6995159596122E-93
**Minimum RMSD**	AlphaFold 3 vs AutoDock4 (Rigid)	485	1097.5	3.00195464728952E-78	1.68554359163324E-82	9.90645033605542E-77	5.73084821155303E-81
**Minimum RMSD**	AlphaFold 3 vs DOCK6 (Rigid)	276	12.0	5.85488849318285E-47	4.7515166405335E-53	1.05387992877291E-45	1.23539432653871E-51
**Minimum RMSD**	AlphaFold 3 vs DiffDock	412	8618.5	1.08894264368932E-44	1.28361237032292E-10	1.85120249427184E-43	2.43886350361354E-09
**Minimum RMSD**	AlphaFold 3 vs GNINA 1.3 (Flexible)	465	274.0	3.84514073921458E-77	1.63084096250264E-90	1.19199362915652E-75	6.19719565751003E-89
**Minimum RMSD**	AlphaFold 3 vs GNINA 1.3 (Rigid)	468	1080.0	2.04301550304586E-75	1.32415451421996E-86	5.72044340852839E-74	4.63454079976988E-85
**Minimum RMSD**	Boltz-2 vs AutoDock Vina (Flexible)	390	1193.0	1.00472539718039E-61	7.02719768466742E-68	2.10992333407881E-60	2.1784312822469E-66
**Minimum RMSD**	Boltz-2 vs AutoDock Vina (Rigid)	388	1673.0	7.99549574317404E-60	1.84508476107567E-67	1.51914419120307E-58	5.53525428322701E-66
**Minimum RMSD**	Boltz-2 vs AutoDock4 (Flexible)	394	55.0	3.96931148535878E-66	1.58464382589426E-73	9.52634756486106E-65	5.22932462545105E-72
**Minimum RMSD**	Boltz-2 vs AutoDock4 (Rigid)	404	1705.5	1.51286572433458E-62	3.54598468898967E-64	3.32830459353609E-61	9.92875712917108E-63
**Minimum RMSD**	Boltz-2 vs DOCK6 (Rigid)	234	17.0	4.79974521611509E-40	2.86137625240869E-41	7.67959234578414E-39	7.15344063102173E-40
**Minimum RMSD**	Boltz-2 vs DiffDock	342	12861.5	2.29168977149753E-19	0.006716645502620930	2.29168977149753E-18	0.05373316402096740
**Minimum RMSD**	Boltz-2 vs GNINA 1.3 (Flexible)	385	290.0	7.36185925409369E-64	4.09973572238393E-68	1.69322762844155E-62	1.31191543116286E-66
**Minimum RMSD**	Boltz-2 vs GNINA 1.3 (Rigid)	390	1479.0	8.43690886785261E-61	7.19380748437883E-65	1.68738177357052E-59	2.08620417046986E-63

**Minimum RMSD**	AutoDock Vina (Flexible) vs AutoDock Vina (Rigid)	938	166264.5	1.49897545246494E-10	1.71627356359427E-05	1.04928281672546E-09	0.00025744103453914100
**Minimum RMSD**	AutoDock Vina (Flexible) vs AutoDock4 (Flexible)	920	67278.5	6.89164487014088E-72	3.63579552029954E-05	1.79182766623663E-70	0.00047265341763894000
**Minimum RMSD**	AutoDock Vina (Flexible) vs AutoDock4 (Rigid)	937	134329.0	6.63828495221964E-25	6.85256886979644E-11	7.96594194266356E-24	1.37051377395929E-09
**Minimum RMSD**	AutoDock Vina (Flexible) vs DOCK6 (Rigid)	531	39029.5	4.1954834806577E-19	1.0	3.77593513259193E-18	1.0
**Minimum RMSD**	AutoDock Vina (Flexible) vs DiffDock	820	9272.0	1.66663790160908E-121	5.38350707042865E-126	7.16654297691905E-120	2.31490804028432E-124
**Minimum RMSD**	AutoDock Vina (Flexible) vs GNINA 1.3 (Flexible)	927	62881.5	9.87688087213635E-78	0.5707503880581740	3.16060187908363E-76	1.0
**Minimum RMSD**	AutoDock Vina (Flexible) vs GNINA 1.3 (Rigid)	936	154055.0	3.24051381112598E-15	1.59690259807905E-05	2.59241104890078E-14	0.0002555044156926480
**Minimum RMSD**	AutoDock Vina (Rigid) vs AutoDock4 (Flexible)	916	59585.0	1.13047237959727E-78	2.53416519803096E-12	3.84360609063072E-77	5.82857995547121E-11
**Minimum RMSD**	AutoDock Vina (Rigid) vs AutoDock4 (Rigid)	935	187212.0	0.00013181266656654200	0.02685669550752440	0.0003954379996996260	0.16339541409023600
**Minimum RMSD**	AutoDock Vina (Rigid) vs DOCK6 (Rigid)	531	30861.0	2.56521417930242E-29	0.0004985148935752060	3.84782126895363E-28	0.004908786448402840

**Minimum RMSD**	AutoDock Vina (Rigid) vs DiffDock	820	12344.0	6.23879453744272E-117	7.87642181679731E-119	2.55790576035152E-115	3.2293329448869E-117
**Minimum RMSD**	AutoDock Vina (Rigid) vs GNINA 1.3 (Flexible)	927	57380.5	2.58483490831266E-83	8.78900501283603E-06	9.30540566992559E-82	0.00014941308521821300
**Minimum RMSD**	AutoDock Vina (Rigid) vs GNINA 1.3 (Rigid)	935	199249.5	0.01799574974085260	1.0	0.03599149948170510	1.0
**Minimum RMSD**	AutoDock4 (Flexible) vs AutoDock4 (Rigid)	952	46041.5	1.08185727396308E-100	2.13728697845071E-17	4.21924336845602E-99	5.1294887482817E-16
**Minimum RMSD**	AutoDock4 (Flexible) vs DOCK6 (Rigid)	537	57585.0	4.69861407236119E-05	0.02334220201289090	0.0001879445628944470	0.16339541409023600
**Minimum RMSD**	AutoDock4 (Flexible) vs DiffDock	800	2944.0	7.90487386874854E-128	3.74720094794675E-128	3.55719324093685E-126	1.68624042657604E-126
**Minimum RMSD**	AutoDock4 (Flexible) vs GNINA 1.3 (Flexible)	909	161123.5	8.00256107239533E-09	0.00030059760744045100	4.8015366434372E-08	0.0033065736818449600
**Minimum RMSD**	AutoDock4 (Flexible) vs GNINA 1.3 (Rigid)	916	61991.0	3.09351547821565E-76	2.53416519803096E-12	9.28054643464696E-75	5.82857995547121E-11
**Minimum RMSD**	AutoDock4 (Rigid) vs DOCK6 (Rigid)	553	34595.5	5.62157458405082E-29	3.0908593764592E-05	7.87020441767115E-28	0.0004327203127042880
**Minimum RMSD**	AutoDock4 (Rigid) vs DiffDock	819	14705.5	2.74287628591507E-113	3.92451592709842E-115	1.09715051436603E-111	1.56980637083937E-113
**Minimum RMSD**	AutoDock4 (Rigid) vs GNINA 1.3 (Flexible)	926	50340.0	1.5605860823162E-90	6.22018847409737E-11	5.93022711280154E-89	1.30623957956045E-09
**Minimum RMSD**	AutoDock4 (Rigid) vs GNINA 1.3 (Rigid)	935	205754.5	0.1145274595318600	0.03400640286438700	0.1145274595318600	0.17003201432193500
**Minimum RMSD**	DOCK6 (Rigid) vs DiffDock	425	1116.0	5.46301869352176E-68	3.6930404900643E-64	1.36575467338044E-66	9.97120932317361E-63

**Minimum RMSD**	DOCK6 (Rigid) vs GNINA 1.3 (Flexible)	524	50464.5	1.28851105985526E-07	1.0	6.44255529927631E-07	1.0
**Minimum RMSD**	DOCK6 (Rigid) vs GNINA 1.3 (Rigid)	532	32128.0	8.55038848231076E-28	0.00012604645012081400	1.1115505027004E-26	0.0015125574014497700
**Minimum RMSD**	DiffDock vs GNINA 1.3 (Flexible)	813	4516.0	1.47095725439058E-127	6.05855094247383E-128	6.47221191931855E-126	2.66576241468848E-126
**Minimum RMSD**	DiffDock vs GNINA 1.3 (Rigid)	821	12408.0	5.0686022875727E-117	2.36533412838596E-120	2.12881296078053E-115	9.93440333922102E-119
**Minimum RMSD**	GNINA 1.3 (Flexible) vs GNINA 1.3 (Rigid)	928	54990.0	5.13421525605617E-86	1.85771786545522E-06	1.89965964474078E-84	3.3438921578194E-05
**Minimum RMSD**	AlphaFold 3 vs Boltz-2	407	18190.5	9.06591175609152E-23	0.036713856362704100	8.15932058048237E-22	0.25699699453892900
**Minimum RMSD**	AlphaFold 3 vs AutoDock Vina (Flexible)	469	930.0	5.38065563221258E-76	7.08334995259276E-85	1.6680032459859E-74	2.62083948245932E-83
**Minimum RMSD**	AlphaFold 3 vs AutoDock Vina (Rigid)	469	1032.0	1.02248799948758E-75	3.06099285675173E-84	3.06746399846274E-74	1.10195742843062E-82
**Minimum RMSD**	AlphaFold 3 vs AutoDock4 (Flexible)	474	19.0	2.51299617454684E-79	2.94598742109445E-97	8.54418699345926E-78	1.14893509422684E-95
**Minimum RMSD**	AlphaFold 3 vs AutoDock4 (Rigid)	485	1625.5	7.33768947388953E-77	3.60603149397091E-76	2.42143752638354E-75	1.15393007807069E-74
**Minimum RMSD**	AlphaFold 3 vs DOCK6 (Rigid)	276	14.0	5.98399189694543E-47	1.41485193908366E-54	1.07711854145018E-45	3.67861504161751E-53
**Minimum RMSD**	AlphaFold 3 vs DiffDock	412	9444.5	1.26651380182014E-42	0.0006067668456360640	1.7731193225482E-41	0.006067668456360640
**Minimum RMSD**	AlphaFold 3 vs GNINA 1.3 (Flexible)	465	461.0	1.27717837904437E-76	1.33074142746439E-91	4.08697081294199E-75	5.05681742436469E-90

**Minimum RMSD**	AlphaFold 3 vs GNINA 1.3 (Rigid)	468	1075.5	1.98591448638961E-75	3.60004397318536E-78	5.75915201052986E-74	1.22401495088302E-76
**Minimum RMSD**	Boltz-2 vs AutoDock Vina (Flexible)	390	2151.0	1.1735599167135E-58	3.04630337898371E-67	2.58183181676971E-57	9.13891013695114E-66
**Minimum RMSD**	Boltz-2 vs AutoDock Vina (Rigid)	388	2320.0	9.23932189154812E-58	1.37105160343757E-66	1.94025759722511E-56	3.97604964996895E-65
**Minimum RMSD**	Boltz-2 vs AutoDock4 (Flexible)	394	35.0	3.40813794765531E-66	5.69488791741111E-79	8.86115866390381E-65	1.99321077109389E-77
**Minimum RMSD**	Boltz-2 vs AutoDock4 (Rigid)	404	2811.0	3.6002797690533E-59	6.8413743000137E-60	8.64067144572793E-58	1.8471710610037E-58
**Minimum RMSD**	Boltz-2 vs DOCK6 (Rigid)	234	93.0	1.27109485043738E-39	2.42323594513378E-44	1.6524233055686E-38	6.05808986283445E-43
**Minimum RMSD**	Boltz-2 vs DiffDock	342	12589.5	5.8544908974631E-20	0.2912928476178100	4.09814362822417E-19	1.0
**Minimum RMSD**	Boltz-2 vs GNINA 1.3 (Flexible)	385	1054.0	2.57884602037244E-61	3.6677503873187E-71	6.4471150509311E-60	1.1370026200688E-69
**Minimum RMSD**	Boltz-2 vs GNINA 1.3 (Rigid)	390	2767.0	9.99156053249676E-57	2.11956404112796E-60	1.99831210649935E-55	5.9347793151583E-59
**Minimum RMSD**	AutoDock Vina (Flexible) vs AutoDock Vina (Rigid)	938	98059.0	5.10163715998106E-49	1.89203994546935E-09	9.69311060396401E-48	2.64885592365709E-08
**Minimum RMSD**	AutoDock Vina (Flexible) vs AutoDock4 (Flexible)	920	27705.0	1.90098384791078E-115	1.56222562156721E-11	7.0336402372699E-114	2.65578355666426E-10
**Minimum RMSD**	AutoDock Vina (Flexible) vs AutoDock4 (Rigid)	937	101090.0	1.713552629246E-46	2.10957702988885E-13	2.91303946971821E-45	3.79723865379994E-12
**Minimum RMSD**	AutoDock Vina (Flexible) vs DOCK6 (Rigid)	531	38768.0	2.14453907358045E-19	0.15472892348537400	1.28672344414827E-18	0.9283735409122460

**Minimum RMSD**	AutoDock Vina (Flexible) vs DiffDock	820	6007.5	1.83909781164577E-126	3.21843786103216E-141	7.54030102774764E-125	1.38392828024383E-139
**Minimum RMSD**	AutoDock Vina (Flexible) vs GNINA 1.3 (Flexible)	927	174568.5	6.82679417226943E-07	0.00808525359270735	3.41339708613472E-06	0.07276728233436620
**Minimum RMSD**	AutoDock Vina (Flexible) vs GNINA 1.3 (Rigid)	936	141971.5	9.46696843826928E-21	9.08852729332585E-11	7.57357475061543E-20	1.45416436693214E-09
**Minimum RMSD**	AutoDock Vina (Rigid) vs AutoDock4 (Flexible)	916	12763.0	6.92859773441587E-134	2.41529513373351E-25	3.04858300314298E-132	5.31364929421372E-24
**Minimum RMSD**	AutoDock Vina (Rigid) vs AutoDock4 (Rigid)	935	215784.0	0.715912695516763	0.2884874633234860	0.715912695516763	1.0
**Minimum RMSD**	AutoDock Vina (Rigid) vs DOCK6 (Rigid)	531	20488.0	1.33679297851741E-45	1.93656723639474E-08	2.13886876562786E-44	2.32388068367369E-07
**Minimum RMSD**	AutoDock Vina (Rigid) vs DiffDock	820	9913.0	1.525531551399E-120	1.46447091450056E-127	5.94957305045609E-119	6.00433074945232E-126
**Minimum RMSD**	AutoDock Vina (Rigid) vs GNINA 1.3 (Flexible)	927	72934.0	4.85659743435893E-68	1.44769019228053E-14	1.31128130727691E-66	2.75061136533302E-13
**Minimum RMSD**	AutoDock Vina (Rigid) vs GNINA 1.3 (Rigid)	935	192751.0	0.0019297906489666500	0.5005813519490320	0.0057893719468999950	1.0
**Minimum RMSD**	AutoDock4 (Flexible) vs AutoDock4 (Rigid)	952	14615.5	5.32690123265371E-138	2.9709841062429E-29	2.39710555469417E-136	7.13036185498296E-28
**Minimum RMSD**	AutoDock4 (Flexible) vs DOCK6 (Rigid)	537	56920.5	2.09183990652475E-05	0.009521891184098850	8.36735962609899E-05	0.07617512947279080

**Minimum RMSD**	AutoDock4 (Flexible) vs DiffDock	800	222.0	3.19114811011997E-132	4.0470783689004E-150	1.37219368735159E-130	1.82118526600518E-148
**Minimum RMSD**	AutoDock4 (Flexible) vs GNINA 1.3 (Flexible)	909	51811.5	2.5775147793932E-85	6.01051628492324E-06	9.02130172787621E-84	6.61156791341556E-05
**Minimum RMSD**	AutoDock4 (Flexible) vs GNINA 1.3 (Rigid)	916	27458.0	5.80438178769128E-115	2.76669844021685E-27	2.08957744356886E-113	6.36340641249876E-26
**Minimum RMSD**	AutoDock4 (Rigid) vs DOCK6 (Rigid)	553	23330.5	1.44090468361138E-45	1.96023530744967E-09	2.16135702541707E-44	2.64885592365709E-08
**Minimum RMSD**	AutoDock4 (Rigid) vs DiffDock	819	10521.0	1.87759387296463E-119	5.68460285570144E-125	7.13485671726558E-118	2.27384114228058E-123
**Minimum RMSD**	AutoDock4 (Rigid) vs GNINA 1.3 (Flexible)	926	82879.0	6.97642434600262E-59	1.48338025340695E-18	1.6045775995806E-57	3.11509853215459E-17
**Minimum RMSD**	AutoDock4 (Rigid) vs GNINA 1.3 (Rigid)	935	195415.5	0.004656610653496070	0.6850706623180770	0.009313221306992150	1.0
**Minimum RMSD**	DOCK6 (Rigid) vs DiffDock	425	142.0	6.12444718822463E-71	2.33709739053016E-76	1.7148452127029E-69	7.71242138874954E-75
**Minimum RMSD**	DOCK6 (Rigid) vs GNINA 1.3 (Flexible)	524	34019.5	1.20825179044835E-23	1.0	1.20825179044835E-22	1.0
**Minimum RMSD**	DOCK6 (Rigid) vs GNINA 1.3 (Rigid)	532	27597.5	2.94715134630361E-34	1.85615451492803E-10	3.24186648093397E-33	2.78423177239205E-09
**Minimum RMSD**	DiffDock vs GNINA 1.3 (Flexible)	813	3719.0	8.33228143361976E-129	1.6374816167633E-147	3.4995582021203E-127	7.20491911375854E-146
**Minimum RMSD**	DiffDock vs GNINA 1.3 (Rigid)	821	9389.0	1.65128757866696E-121	8.87687087636959E-128	6.60515031466783E-120	3.72828576807523E-126
**Minimum RMSD**	GNINA 1.3 (Flexible) vs GNINA 1.3 (Rigid)	928	108334.5	2.38016076967261E-39	1.35440451754294E-16	2.85619292360713E-38	2.70880903508589E-15

Table S6. Redocking performance summary statistics across docking/scoring tools: sample sizes, failed-run counts, RMSD distributions (mean ± SD, median [IQR]), and success rates (RMSD ≤ 2.0 Å) with 95% Wilson confidence intervals, stratified by minimum-energy and minimum-RMSD pose selection.

Aggregation Type	Tool	Expected Total (N)	Valid Evaluated (n)	Failed Runs	Failure Rate (%)	Mean ± SD (Å)	Standard Error (Å)	Median (IQR) (Å)	Success Rate (≤ 2.0 Å)	95% Wilson CI
**Minimum RMSD**	AutoDock Vina (Flexible)	494	488	6	1.21%	7.20 ± 3.38	0.15	7.01 (2.65)	3.07%	[1.9%, 5.0%]
**Minimum RMSD**	AutoDock Vina (Rigid)	494	487	7	1.42%	4.54 ± 4.24	0.19	3.77 (6.34)	41.07%	[36.8%, 45.5%]
**Minimum RMSD**	AutoDock4 (Flexible)	494	478	16	3.24%	6.50 ± 2.58	0.12	6.35 (2.82)	0.84%	[0.3%, 2.1%]
**Minimum RMSD**	AutoDock4 (Rigid)	494	488	6	1.21%	4.99 ± 4.32	0.20	4.36 (6.30)	34.84%	[30.7%, 39.2%]
**Minimum RMSD**	DOCK6 (Rigid)	494	473	21	4.25%	3.45 ± 3.87	0.18	1.52 (5.38)	56.45%	[51.9%, 60.8%]
**Minimum RMSD**	DiffDock	494	421	73	14.78%	1.44 ± 2.14	0.10	0.84 (0.68)	87.41%	[83.9%, 90.2%]
**Minimum RMSD**	GNINA 1.3 (Flexible)	494	475	19	3.85%	12.28 ± 4.40	0.20	12.83 (5.42)	1.68%	[0.9%, 3.3%]
**Minimum RMSD**	GNINA 1.3 (Rigid)	494	481	13	2.63%	2.19 ± 3.54	0.16	0.99 (1.67)	73.18%	[69.0%, 76.9%]
**Minimum RMSD**	AutoDock Vina (Flexible)	494	488	6	1.21%	4.60 ± 3.09	0.14	4.41 (2.41)	11.89%	[9.3%, 15.1%]
**Minimum RMSD**	AutoDock Vina (Rigid)	494	487	7	1.42%	2.80 ± 3.35	0.15	1.60 (3.09)	58.32%	[53.9%, 62.6%]
**Minimum RMSD**	AutoDock4 (Flexible)	494	478	16	3.24%	4.08 ± 2.19	0.10	4.01 (1.57)	5.44%	[3.7%, 7.9%]
**Minimum RMSD**	AutoDock4 (Rigid)	494	488	6	1.21%	3.03 ± 3.34	0.15	1.85 (3.64)	52.05%	[47.6%, 56.4%]
**Minimum RMSD**	DOCK6 (Rigid)	494	473	21	4.25%	2.52 ± 3.18	0.15	0.94 (3.14)	65.75%	[61.4%, 69.9%]
**Minimum RMSD**	DiffDock	494	421	73	14.78%	1.06 ± 1.08	0.05	0.74 (0.60)	90.74%	[87.6%, 93.1%]
**Minimum RMSD**	GNINA 1.3 (Flexible)	494	475	19	3.85%	6.40 ± 3.60	0.17	5.84 (4.01)	6.32%	[4.5%, 8.9%]

**Minimum RMSD**	GNINA 1.3 (Rigid)	494	481	13	2.63%	1.36 ± 2.84	0.13	0.78 (0.84)	87.73%	[84.5%, 90.4%]

Table S7. Pairwise statistical comparisons of redocking performance between tools: Wilcoxon signed-rank tests (paired RMSD differences) and McNemar's tests (paired success/failure outcomes), with Holm–Bonferroni-adjusted p-values for multiple comparisons.

Aggregation Type	Comparison	Matched Pairs (n)	Wilcoxon W	Wilcoxon p-value	McNemar p-value	Wilcoxon p-value (Holm-adjusted)	McNemar p-value (Holm-adjusted)
**Minimum RMSD**	AutoDock Vina (Flexible) vs AutoDock Vina (Rigid)	487	21858.0	1.24153131726716E-33	1.92509818098389E-40	1.36568444899388E-32	3.08015708957422E-39
**Minimum RMSD**	AutoDock Vina (Flexible) vs AutoDock4 (Flexible)	478	42088.0	5.30929941666744E-07	0.015293371030198900	2.12371976666697E-06	0.061173484120795800
**Minimum RMSD**	AutoDock Vina (Flexible) vs AutoDock4 (Rigid)	487	28986.0	1.21022999520609E-22	8.47616502831578E-32	1.08920699568548E-21	9.32378153114736E-31
**Minimum RMSD**	AutoDock Vina (Flexible) vs DOCK6 (Rigid)	471	14824.0	4.87030937308925E-43	1.88950356734175E-54	7.30546405963387E-42	3.7790071346835E-53
**Minimum RMSD**	AutoDock Vina (Flexible) vs DiffDock	417	1375.0	7.86382906747075E-66	6.98689527708568E-78	1.65140410416886E-64	1.88646172481313E-76
**Minimum RMSD**	AutoDock Vina (Flexible) vs GNINA 1.3 (Flexible)	473	6349.5	1.10865389444062E-62	0.19043026382552000	1.99557700999312E-61	0.38086052765104100
**Minimum RMSD**	AutoDock Vina (Flexible) vs GNINA 1.3 (Rigid)	479	3370.5	2.7997478175649E-71	2.43886623157283E-73	7.27934432566874E-70	5.60939233261751E-72

**Minimum RMSD**	AutoDock Vina (Rigid) vs AutoDock4 (Flexible)	477	25432.0	1.05734365596511E-25	1.13727304942925E-43	1.05734365596511E-24	2.16081879391557E-42
**Minimum RMSD**	AutoDock Vina (Rigid) vs AutoDock4 (Rigid)	487	49749.0	0.0018676824455007400	0.027408945115716300	0.0037353648910014900	0.082226835347149
**Minimum RMSD**	AutoDock Vina (Rigid) vs DOCK6 (Rigid)	471	41321.5	1.40911694736444E-06	1.16279286616318E-07	4.22735084209333E-06	6.97675719697908E-07
**Minimum RMSD**	AutoDock Vina (Rigid) vs DiffDock	417	12970.0	1.82958174774105E-35	1.95009480082593E-34	2.37845627206336E-34	2.73013272115631E-33
**Minimum RMSD**	AutoDock Vina (Rigid) vs GNINA 1.3 (Flexible)	472	2189.0	4.06848600472469E-73	7.49691065270055E-41	1.09849122127567E-71	1.27447481095909E-39
**Minimum RMSD**	AutoDock Vina (Rigid) vs GNINA 1.3 (Rigid)	479	20095.5	5.95142923134493E-35	7.32246981041564E-28	7.14171507761392E-34	7.32246981041564E-27
**Minimum RMSD**	AutoDock4 (Flexible) vs AutoDock4 (Rigid)	477	31555.0	2.96090475750798E-17	6.65871092591556E-37	2.07263333025559E-16	9.98806638887334E-36
**Minimum RMSD**	AutoDock4 (Flexible) vs DOCK6 (Rigid)	461	15852.0	5.17727822399328E-39	7.60324980049448E-58	7.2481895135906E-38	1.67271495610879E-56
**Minimum RMSD**	AutoDock4 (Flexible) vs DiffDock	408	1399.0	3.39572051377581E-64	5.69488791741111E-79	6.45186897617404E-63	1.59456861687511E-77
**Minimum RMSD**	AutoDock4 (Flexible) vs GNINA 1.3 (Flexible)	463	4358.0	8.59283417157074E-66	0.34278171114790900	1.71856683431415E-64	0.38086052765104100
**Minimum RMSD**	AutoDock4 (Flexible) vs GNINA 1.3 (Rigid)	469	4118.5	1.58471067196019E-67	1.03985411606559E-75	3.80330561270446E-66	2.59963529016398E-74
**Minimum RMSD**	AutoDock4 (Rigid) vs DOCK6 (Rigid)	472	32549.0	4.26870903213535E-15	2.34818009991765E-13	2.56122541928121E-14	1.87854407993412E-12

**Minimum RMSD**	AutoDock4 (Rigid) vs DiffDock	418	9182.5	1.53521151472834E-44	5.744932200772E-43	2.60985957503818E-43	1.03408779613896E-41
**Minimum RMSD**	AutoDock4 (Rigid) vs GNINA 1.3 (Flexible)	473	4592.0	4.63311470785485E-67	2.46165445258918E-33	1.06561638280662E-65	2.95398534310702E-32
**Minimum RMSD**	AutoDock4 (Rigid) vs GNINA 1.3 (Rigid)	480	15339.5	3.69490507309529E-44	9.77941099401167E-34	5.91184811695246E-43	1.27132342922152E-32
**Minimum RMSD**	DOCK6 (Rigid) vs DiffDock	405	21063.0	1.83889766043861E-17	3.34908528447858E-23	1.47111812835089E-16	3.01417675603072E-22
**Minimum RMSD**	DOCK6 (Rigid) vs GNINA 1.3 (Flexible)	464	3090.0	2.66375436654659E-69	1.0654951069917E-55	6.65938591636647E-68	2.23753972468258E-54
**Minimum RMSD**	DOCK6 (Rigid) vs GNINA 1.3 (Rigid)	472	36443.5	6.4359016499058E-11	4.55019320363173E-09	3.2179508249529E-10	3.18513524254221E-08
**Minimum RMSD**	DiffDock vs GNINA 1.3 (Flexible)	410	703.0	1.05226800851546E-66	5.01933785033399E-77	2.31498961873402E-65	1.30502784108684E-75
**Minimum RMSD**	DiffDock vs GNINA 1.3 (Rigid)	412	36469.0	0.012079027547980600	7.15111767906766E-06	0.012079027547980600	3.57555883953383E-05
**Minimum RMSD**	GNINA 1.3 (Flexible) vs GNINA 1.3 (Rigid)	472	473.0	9.48265734499067E-78	7.72781839227703E-75	2.65514405659739E-76	1.85467641414649E-73
**Minimum RMSD**	AutoDock Vina (Flexible) vs AutoDock Vina (Rigid)	487	20822.0	2.0322435902806E-35	4.87282873163251E-47	3.0483653854209E-34	7.79652597061202E-46
**Minimum RMSD**	AutoDock Vina (Flexible) vs AutoDock4 (Flexible)	478	45280.5	7.55126344853942E-05	0.00013572250908123800	0.00030205053794157 70	0.00081433505448742 80
**Minimum RMSD**	AutoDock Vina (Flexible) vs AutoDock4 (Rigid)	487	27283.0	4.60783659166084E-25	1.26895373992155E-37	4.14705293249476E-24	1.77653523589017E-36

**Minimum RMSD**	AutoDock Vina (Flexible) vs DOCK6 (Rigid)	471	22054.0	8.04325386614379E-30	2.34423077698645E-50	9.65190463937254E-29	4.21961539857562E-49
**Minimum RMSD**	AutoDock Vina (Flexible) vs DiffDock	417	1901.0	3.02563184904021E-64	5.77706001228874E-71	7.26151643769651E-63	1.32872380282641E-69
**Minimum RMSD**	AutoDock Vina (Flexible) vs GNINA 1.3 (Flexible)	473	21583.5	4.73202197994635E-31	0.0010540340457847900	6.15162857393025E-30	0.0052701702289239300
**Minimum RMSD**	AutoDock Vina (Flexible) vs GNINA 1.3 (Rigid)	479	1726.0	1.45849646578207E-75	5.11307328844127E-79	3.93794045761159E-74	1.32939905499473E-77
**Minimum RMSD**	AutoDock Vina (Rigid) vs AutoDock4 (Flexible)	477	23306.0	4.73636909692593E-29	2.5452031623434E-55	5.21000600661853E-28	5.09040632468679E-54
**Minimum RMSD**	AutoDock Vina (Rigid) vs AutoDock4 (Rigid)	487	51652.0	0.0124875629550513000	0.028248548589406100	0.0249751259101025000	0.08474564576821820
**Minimum RMSD**	AutoDock Vina (Rigid) vs DOCK6 (Rigid)	471	47673.0	0.0074802816862402900	0.013393313038997700	0.0224408450587209000	0.05357325215599090
**Minimum RMSD**	AutoDock Vina (Rigid) vs DiffDock	417	14779.5	1.3720514668288E-31	3.3001218153313E-25	1.92087205356033E-30	3.3001218153313E-24
**Minimum RMSD**	AutoDock Vina (Rigid) vs GNINA 1.3 (Flexible)	472	8160.5	3.97828777511506E-58	1.54212858206437E-51	8.75223310525313E-57	2.9300443059223E-50
**Minimum RMSD**	AutoDock Vina (Rigid) vs GNINA 1.3 (Rigid)	479	15733.0	3.69725619201195E-43	1.50127545864915E-27	6.65506114562151E-42	1.65140300451407E-26
**Minimum RMSD**	AutoDock4 (Flexible) vs AutoDock4 (Rigid)	477	27980.5	5.69965251636034E-22	8.62367651890238E-49	4.55972201308827E-21	1.4660250082134E-47

**Minimum RMSD**	AutoDock4 (Flexible) vs DOCK6 (Rigid)	461	22034.0	1.08422921308787E-27	3.49657832740033E-60	1.08422921308787E-26	7.69247232028072E-59
**Minimum RMSD**	AutoDock4 (Flexible) vs DiffDock	408	1667.5	2.28400013307813E-63	1.55198089876459E-75	5.25320030607969E-62	3.87995224691147E-74
**Minimum RMSD**	AutoDock4 (Flexible) vs GNINA 1.3 (Flexible)	463	14851.0	1.81103171081457E-41	0.47048642205879000	3.07875390838477E-40	0.9409728441175790
**Minimum RMSD**	AutoDock4 (Flexible) vs GNINA 1.3 (Rigid)	469	1758.0	9.53183858416296E-74	9.83155284318004E-86	2.47827803188237E-72	2.75283479609041E-84
**Minimum RMSD**	AutoDock4 (Rigid) vs DOCK6 (Rigid)	472	42686.5	9.52792060500108E-06	2.5340149271215E-06	4.76396030250054E-05	1.77381044898505E-05
**Minimum RMSD**	AutoDock4 (Rigid) vs DiffDock	418	12683.0	2.5629607420115E-36	1.44540323174472E-31	4.1007371872184E-35	1.73448387809367E-30
**Minimum RMSD**	AutoDock4 (Rigid) vs GNINA 1.3 (Flexible)	473	9582.5	5.0746957762427E-55	8.54461218856294E-47	1.06568611301097E-53	1.28169182828444E-45
**Minimum RMSD**	AutoDock4 (Rigid) vs GNINA 1.3 (Rigid)	480	14618.0	1.293201325753E-45	1.22616633053079E-31	2.45708251893069E-44	1.59401622969002E-30
**Minimum RMSD**	DOCK6 (Rigid) vs DiffDock	405	23804.0	3.29560817807443E-13	3.94227631872567E-19	2.3069257246521E-12	3.54804868685311E-18
**Minimum RMSD**	DOCK6 (Rigid) vs GNINA 1.3 (Flexible)	464	11538.0	9.6978410336818E-49	1.65063271064971E-57	1.93956820673636E-47	3.4663286923644E-56
**Minimum RMSD**	DOCK6 (Rigid) vs GNINA 1.3 (Rigid)	472	35066.0	2.59925645919176E-12	1.03550045113911E-16	1.55955387551506E-11	8.28400360911288E-16
**Minimum RMSD**	DiffDock vs GNINA 1.3 (Flexible)	410	1028.0	1.086041388119E-65	7.2983871543247E-75	2.71510347029749E-64	1.75161291703793E-73
**Minimum RMSD**	DiffDock vs GNINA 1.3 (Rigid)	412	41734.5	0.7394021629775200	0.5319710580974010	0.7394021629775200	0.9409728441175790

**Minimum RMSD**	GNINA 1.3 (Flexible) vs GNINA 1.3 (Rigid)	472	1096.5	4.75228462604127E-76	5.25812002694894E-84	1.33063969529156E-74	1.41969240727621E-82

Receptor conformations were selected based on docking performance for each target. Holo structures were used for AChE and BACE1, whereas the apo structure was used for GSK-3β.

Table S8: Descriptive statistics of docking accuracy (RMSD) by tool, aggregation type, and docking condition, with 95% Wilson confidence intervals for success rate (RMSD ≤ 2 Å)

Aggregation Type	Docking Condition	Tool	N	Mean ± SD (Å)	Median (Å)	IQR (Å)	Success Rate (%)	Wilson 95% CI (%)
Minimum RMSD	Redock	DiffDock	73	1.23 ± 1.55	0.70	0.48–1.09	87.67	78.20–93.38
Minimum RMSD	Redock	GNINA 1.3 (Rigid)	126	2.22 ± 2.57	1.09	0.47–3.15	71.43	63.00–78.59
Minimum RMSD	Crossdock	AlphaFold 3	127	1.08 ± 1.71	0.55	0.37–0.92	88.19	81.43–92.71
Minimum RMSD	Crossdock	DiffDock	71	1.92 ± 1.92	1.47	0.93–1.90	78.87	68.03–86.76
Minimum RMSD	Crossdock	GNINA 1.3 (Rigid)	125	6.17 ± 3.67	5.70	3.08–8.32	8.00	4.40–14.10
Minimum RMSD	Crossdock	Boltz-2	95	1.62 ± 1.99	0.87	0.58–1.72	82.11	73.20–88.52
Minimum RMSD	Redock	DiffDock	91	0.84 ± 0.76	0.59	0.42–0.94	93.41	86.35–96.94
Minimum RMSD	Redock	GNINA 1.3 (Rigid)	126	1.11 ± 1.13	0.69	0.46–1.24	87.30	80.37–92.03
Minimum RMSD	Crossdock	AlphaFold 3	127	0.76 ± 1.25	0.39	0.29–0.71	92.13	86.11–95.67
Minimum RMSD	Crossdock	DiffDock	92	1.26 ± 1.39	0.92	0.69–1.46	93.48	86.49–96.98

Minimum RMSD	Crossdock	GNINA 1.3 (Rigid)	125	3.97 ± 2.56	3.19	2.31–4.92	13.60	8.67–20.70
Minimum RMSD	Crossdock	Boltz-2	95	1.07 ± 1.59	0.59	0.40–0.93	89.47	81.70–94.18

Table S9: Pairwise statistical comparisons between docking tools by aggregation type and docking condition (Wilcoxon signed-rank test and McNemar's exact test, Holm-adjusted p-values)

Aggregation Type	Docking Condition	Comparison	Matched Pairs (N)	Wilcoxon W	Wilcoxon P (unadj.)	McNemar P (unadj.)	Wilcoxon P (Holm-adj.)	McNemar P (Holm-adj.)
Minimum RMSD	Redock	DiffDock vs GNINA 1.3 (Rigid)	72	1056.0	0.1477	0.0963	0.1477	0.0963
Minimum RMSD	Crossdock	AlphaFold 3 vs DiffDock	71	374.0	2.22×10^{-7}	0.302	6.66×10^{-7}	0.630
Minimum RMSD	Crossdock	AlphaFold 3 vs GNINA 1.3 (Rigid)	125	130.0	6.51×10^{-21}	5.38×10^{-28}	3.91×10^{-20}	3.23×10^{-27}
Minimum RMSD	Crossdock	AlphaFold 3 vs Boltz-2	94	1224.5	1.44×10^{-4}	0.210	2.88×10^{-4}	0.630
Minimum RMSD	Crossdock	DiffDock vs GNINA 1.3 (Rigid)	71	74.0	5.25×10^{-12}	1.78×10^{-15}	2.10×10^{-11}	7.11×10^{-15}
Minimum RMSD	Crossdock	DiffDock vs Boltz-2	52	498.5	0.0828	0.607	0.0828	0.630
Minimum RMSD	Crossdock	GNINA 1.3 (Rigid) vs Boltz-2	93	138.0	4.32×10^{-15}	4.21×10^{-18}	2.16×10^{-14}	2.11×10^{-17}

Minimum RMSD	Redock	DiffDock vs GNINA 1.3 (Rigid)	90	2026.5	0.933	1.000	0.933	1.000
Minimum RMSD	Crossdock	AlphaFold 3 vs DiffDock	92	615.0	2.95×10^{-9}	1.000	8.85×10^{-9}	1.000
Minimum RMSD	Crossdock	AlphaFold 3 vs GNINA 1.3 (Rigid)	125	66.0	1.44×10^{-21}	2.07×10^{-27}	8.64×10^{-21}	1.24×10^{-26}
Minimum RMSD	Crossdock	AlphaFold 3 vs Boltz-2	94	1121.5	2.80×10^{-5}	0.508	5.59×10^{-5}	1.000
Minimum RMSD	Crossdock	DiffDock vs GNINA 1.3 (Rigid)	92	31.0	2.24×10^{-16}	5.29×10^{-23}	1.12×10^{-15}	2.65×10^{-22}
Minimum RMSD	Crossdock	DiffDock vs Boltz-2	66	662.5	4.66×10^{-3}	0.0313	4.66×10^{-3}	0.0938
Minimum RMSD	Crossdock	GNINA 1.3 (Rigid) vs Boltz-2	93	246.0	1.07×10^{-13}	6.81×10^{-17}	4.30×10^{-13}	2.72×10^{-16}
